# Thermally Activated and Nonactivated Excited State Decay of [Cr(dgpy)_2_]^3+^

**DOI:** 10.1021/acs.inorgchem.5c03668

**Published:** 2025-10-01

**Authors:** Steven Sittel, Robert Naumann, Christoph Förster, Maximilian E. Huber, Jennifer Meyer, Katja Heinze

**Affiliations:** † Department of Chemistry, 9182 Johannes Gutenberg University Mainz, Duesbergweg 10-14, 55128 Mainz, Germany; ‡ Fachbereich Chemie und Forschungszentrum OPTIMAS, 26562 RPTU Kaiserslautern-Landau, Erwin-Schroedinger Str. 52, 67663 Kaiserslautern, Germany

## Abstract

Photoactive and luminescent chromium­(III) complexes are typically coordinated by aromatic N-heterocyclic chelate ligands such as polypyridines. Photoluminescence quantum yields and lifetimes can achieve up to 30% and several milliseconds, respectively. The effect of aliphatic N-donor ligands (guanidines) on the nonradiative decay of emissive doublet states remained, however, underexplored. Here, we describe the preparation, structure, redox chemistry, gas phase stability, and photophysical properties of the complex **[Cr­(dgpy)**_**2**_ **]­[PF**_**6**_ **]**_**3**_ with pyridine and guanidine donors (dgpy = 2,6-diguanidylpyridine). Variable-temperature luminescence spectroscopy, near-infrared overtone spectroscopy as well as quantum chemical calculations identified the major thermally activated and nonactivated decay pathways and inform on future design criteria for photoactive chromium­(III) complexes.

## Graphical abstract

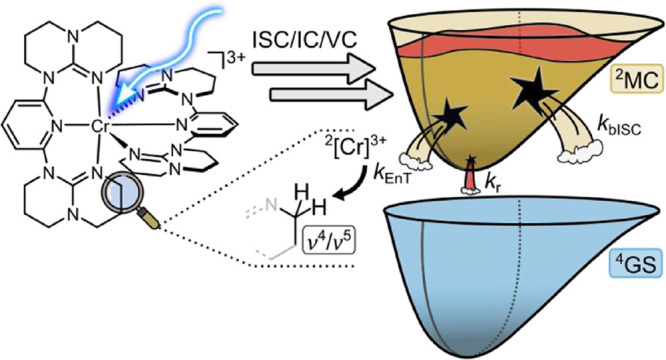

## Introduction

Pseudo-octahedral polypyridine chromium­(III) complexes are notable among photoactive first-row transition metal complexes for their outstanding photophysical and photochemical properties.^1–8^ In a strong ligand field — imposed by chelate ligands forming six-membered rings — chromium­(III) ions have long-lived doublet excited states (^2^MC: ^2^E and ^2^T_1_ in octahedral symmetry) that emit red to near-infrared (NIR) spin-flip (SF) photoluminescence (PL).^2,5,9^ Advances in highly emissive chromium­(III) “molecular ruby” complexes have renewed interest in this area (e.g., [Cr­(L^X^)­(L^Y^)]^3+^ ^10–17^ or [Cr­(dqp)_2_]^3+^,^18–21^ Chart 1), enabling applications in sensing,^22–24^ upconversion,^25,26^ circularly polarized luminescence,^20,21,27–30^ and photocatalysis.^18,19,31–34^ The nephelauxetic effect^10,11,35–37^ has been recognized as key to shifting the SF states to lower energy, for example using cyanide,^38^ carbanionic,^39,40^ amide,^28,41–44^ or carbene ligands^45,46^ or other metal centers with d^3^ or d^2^ electron configuration such as molybdenum­(III),^47^ vanadium­(III),^48,49^ vanadium­(II),^50^ or manganese­(IV).^51,52^

**Chart 1:**
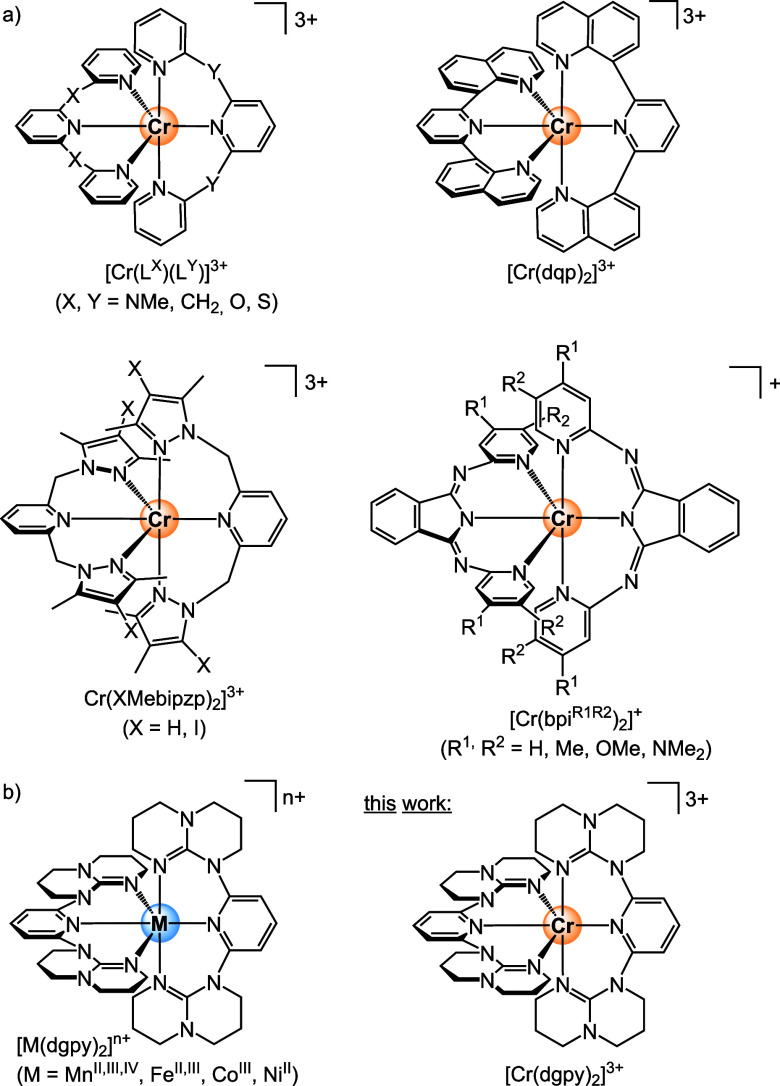
(a) Selected Pseudo-Octahedral Chromium­(III) Complexes with Strong-Field Ligands, but Highly Variable PL Quantum Yields^a^;^10–21,41–43,53^ (b) Homoleptic Metal Complexes of the Ligand dgpy^51,54–57^

a More examples are shown in Chart S1.

The radiative rate constant *k*_r_ for the spin- and Laporte-forbidden SF phosphorescence in pseudo-octahedral chromium­(III) complexes is typically below *k*_r_ < 200 s^–1^ (e.g., Charts 1 and S1).^7^ Hence, the achievable PL quantum yield *Φ* is dominated by the nonradiative rate constant *k*_nr_, which can span several orders of magnitude (10^2^–10^5^ s^–1^, Figure 1a).^7^

**Figure 1:**
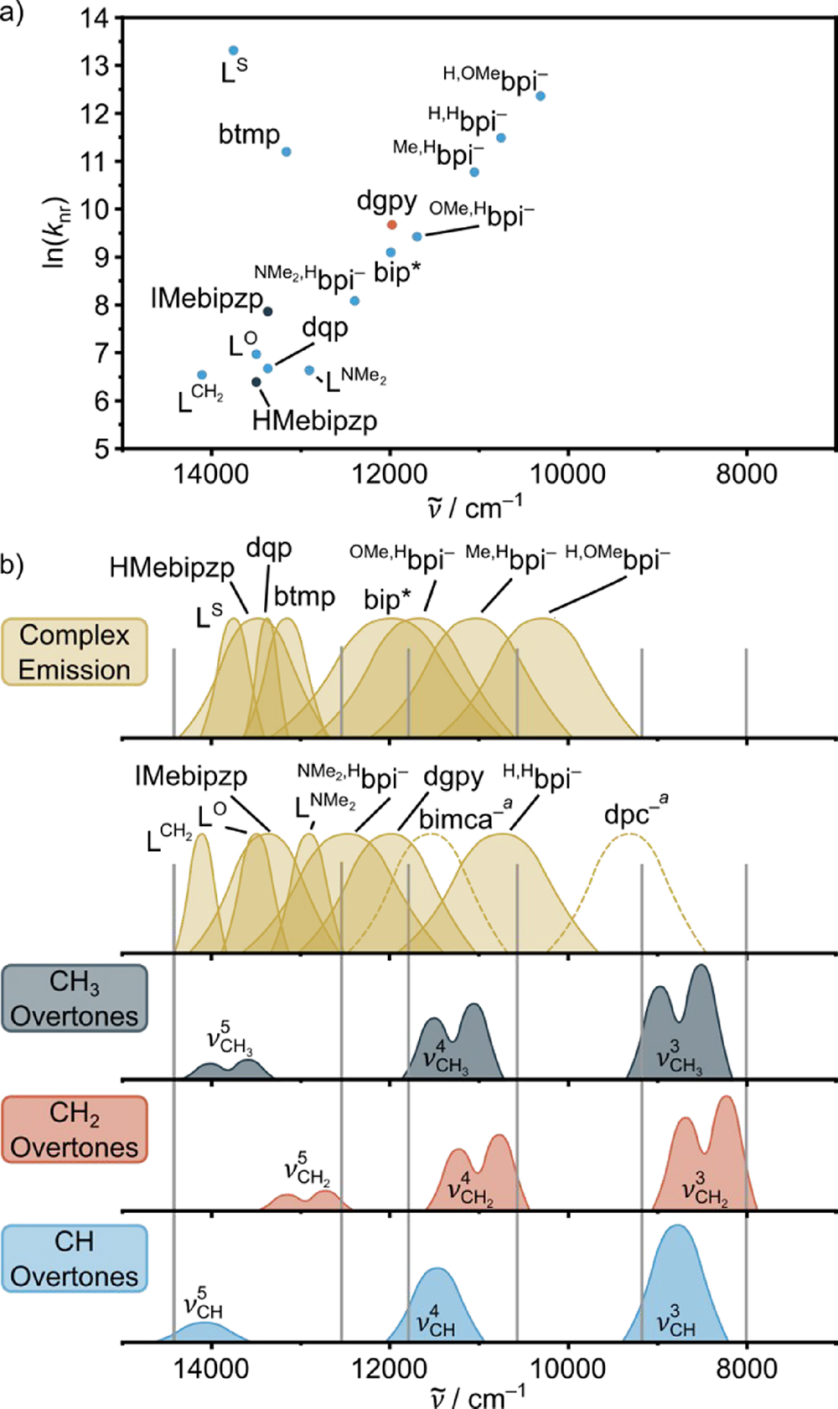
(a) Plot of ln­(*k*_nr_) (at room temperature) versus SF emission energy ν̃ according to eq 1 for various chromium­(III) complexes with the indicated ligands (Charts 1 and S1). (b) Schematic plot of normalized emission bands of homoleptic chromium­(III) complexes with the indicated ligands and C–H overtone absorption bands for CH_3_,^46,66^ CH_2_,^67^ and CH^15^ units. Note that the intensities of the higher C–H overtones decrease much stronger than schematically indicated here, that CH_*n*_ overtone regions additionally can contain combination bands,^67^ that CH_2_ in rings splits into axial and equatorial C–H modes and that CH_3_ overtones can split when attached to rings,^66^ which is qualitatively indicated. ^a^Nonemissive complex at room temperature; data from absorption or 77 K spectrum.

Heavy atoms as substituents can increase both *k*_r_ and *k*_nr_ via the heavy atom effect, i.e. increased spin–orbit coupling. Yet, this increased spin–orbit coupling can affect *k*_r_ and *k*_nr_ to a similar extend so that *Φ* remains constant (X = H → X = I substitution in [Cr­(XMebipzp)_2_]^3+^; Chart 1, Figure 1a).^53^ The heavy atom effect can even favor *k*_nr_ over *k*_r_ (X = O → X = S substitution in [Cr­(L^X^)_2_]^3+^; Chart 1), so that *Φ* even decreases.^10^

Two major nonradiative SF state decay pathways have been proposed. The first process is thermally activated back-intersystem crossing (bISC) from the doublet SF states to distorted quartet metal-centered excited states (^4^MC), which rapidly evolve to the ^4^A_2_ ground state.^2,7^ This decay path depends on the ligand field splitting in the Franck–Condon (FC) geometry and on the flexibility of the ligand sphere to accommodate the distorted ^4^MC state with elongated metal–ligand bonds. Both factors determine the energy of the relaxed lowest-energy ^4^MC state and thus the activation barrier *E*_a_ for the thermally activated bISC ^2^MC →^4^MC.

The second relaxation path of SF excited states of chromium­(III) complexes (weak coupling limit) has been described by the energy gap law (eq 1),^58^ which had been successful for charge transfer excited states (strong coupling limit).^59–62^ While the series of complexes with bpi^R1R2–^ ligands (Chart 1) follows the energy gap relation (eq 1) well,^41^ other Cr complexes do not seem to follow this relation (Figure 1a, see Chart S1 for structures of all complexes).

$$\ln(k_{\mathrm{nr}})\propto -\tilde{\nu }$$ 1

Clearly, a strict dependence of *Φ* or *k*_nr_ on the SF energy ν̃ is not obvious. This applies even to the rather homogeneous class of chromium­(III) emitters with meridional tridentate ligands forming six-membered chelate rings (Charts 1 and S1, Figure 1a). For example, the SF energy ν̃ of chromium­(III) complexes with L^X^ (X = CH_2_, O, NMe), Me-bipzp and dqp ligands cover approximately 1200 cm^–1^, but *k*_nr_ does not change significantly (Figure 1a). Introduction of iodine approximately keeps ν̃, but significantly increases *k*_nr_ (Figure 1a, [Cr­(XMebipzp)_2_]^3+^).^53^ Substitution of X = O by X = S barely changes ν̃ as well, but increases *k*_nr_ by orders of magnitude (Figure 1a, [Cr­(L^X^)_2_]^3+^).

A more specific interpretation of the energy gap law in the weak coupling limit is known as resonant vibrational energy transfer to high-frequency X–H overtones ν_XH_^n^ of the ligand or the solvent, typically C–H, N–H or O–H overtones.^63,64^ Indeed, deuteration of the coordinating ligands has been shown to increase *Φ* by decreasing *k*_nr_, while keeping *k*_*r*_ and ν̃ constant.^14,15,65^ The rate constant for this multiphonon relaxation via resonant energy transfer to high-energy oscillators *k*_nr_ depends on the energy-donor–acceptor distance with *d* ^–6^, an orientation factor κ and the Förster-type spectral overlap integral SOI (eqs 2 and 3).^63,64^ Due to the strong dependence on the distance with *d* ^–6^, only X–H oscillators close to the metal center, i.e. in the first coordination sphere, are efficient energy acceptors. The SOI integrates over the product of the area-normalized emission intensity $I_{\mathrm{norm}}(\tilde{\nu })$ and the extinction coefficient of the respective overtone vibration $\varepsilon_{\mathrm{vib}}(\tilde{\nu })$ scaled by ${\tilde{\nu }}^{-4}$ (eq 3).

$$k_{\mathrm{nr}}\propto \kappa^{2}d^{-6}\;\mathrm{SOI}$$ 2

$$\mathrm{SOI}=\int I_{\mathrm{norm}}(\tilde{\nu })\varepsilon_{\mathrm{vib}}(\tilde{\nu }){\tilde{\nu }}^{-4}d\tilde{\nu }$$ 3

If the SF emission band extends over several X–H overtones, summation of the individual SOIs is required. Due to the scaling with ν̃^–4^ and the fact that the molar absorption coefficient ε_vib_(ν̃) of higher overtones is much smaller, this energy transfer mechanism appears only significantly relevant in the regions of the second to fourth C–H overtones ν_CH_^3^ to ν_CH_^5^ with a strongly decreasing impact at higher overtones. This is schematically illustrated in Figure 1b for CH_3_,^46,66^ CH_2_ ^67^ and CH^15^ overtones and the emission bands of a series of chromium­(III) complexes (Charts 1 and S1). We divided the aliphatic C–H modes in CH_2_ and CH_3_ modes (Figure 1b), as their overtone energies (different anharmonicity) and the number and orientation of C–H units differ, and as CH_2_ units are often encountered in rigid cyclic structures, while CH_3_ substituents are most often free rotors. This might have an additional effect on *k*_nr_ via the orientation factor κ (eq 2).

Interestingly, multiphonon relaxation pathways can also be thermally activated, when higher energy excited SF states that contribute to the nonradiative decay are thermally populated according to a Boltzmann distribution.^10^ In such cases, the emission intensity is temperature-dependent and activation barriers of a few hundred wavenumbers can be extracted by applying Arrhenius’ law. These energy barriers roughly correspond to energy gaps between the lowest SF states.^10^ Additionally, changes of emission band shapes, band widths and band positions with temperature can modify the SOIs and hence *k*_nr_.

In most cases reported so far, the CH moieties closest to the chromium center are the α-C–H units of the coordinating terminal aromatic heterocyclic donors (pyridine, quinoline, indazol, Charts 1 and S1), although methyl substituents at the α-position seem to play a role as well ([Cr­(XMebipzp)_2_]^3+^,^53^ N-heterocyclic carbene complexes,^45,46^ Charts 1a and S1). Methylene units close to the chromium­(III) center have not yet been considered, although these are present for example in the classical 1,2-ethylenediamine chromium­(III) complexes.^3,68^

In the present study, we investigate the influence of guanidine donors on the SF emission energy (nephelauxetic effect) and the effect of methylene groups close to the chromium­(III) center on the nonradiative decay (see Figure 1b for estimated CH_2_ overtones^67^) found in the ligand 2,6-diguanidylpyridine (dgpy^69^) on the SF emission of the chromium­(III) center in the complex **[Cr­(dgpy)**_**2**_ **]** ^**3+**^. Although this adaptive tridentate N-donor ligand had been successfully coordinated to the 3d metals manganese, iron, cobalt and nickel in various oxidation states,^51,54–57^ dgpy complexes with chromium­(III) had not been reported so far (Chart 1b).

## Results and Discussion

### Synthesis and Characterization of [Cr­(dgpy)_2_]­[PF_6_]_3_

The ligand dgpy was obtained by a literature procedure,^69^ separated from the KBr byproduct via extraction with toluene and carefully dried to avoid protonation of the basic guanidine sites (Figure S1), which would hamper complex formation. Coordination of dgpy to chromium­(III) triflate in dry THF, salt metathesis with [*n-*Bu_4_N]­[PF_6_] and recrystallization gave **[Cr­(dgpy)**_**2**_ **]­[PF**_**6**_ **]**_**3**_ as orange crystals in 43% yield (Scheme 1). The six-coordinate chromium­(III) complex is stable under noninert conditions (O_2_, H_2_O). Large but twinned crystals of **[Cr­(dgpy)**_**2**_ **]­[PF**_**6**_ **]**_**3**_ **×1.5 DMF** were obtained by recrystallization from DMF/diethyl ether (Figure S2). SC-XRD confirmed the overall structure (Figure S3a), but twinning and disorder of the counterions and in some of the terminal six-membered rings of the guanidine units precluded a detailed analysis of the bond lengths and angles. However, density functional theory (DFT) calculations (B3LYP, TZVPP, ZORA, CPCM, D3BJ) typically describe the geometry of chromium­(III) complexes well (Figure S3b).^10,11,13,14^ The metrical data for **[Cr(dgpy)**_**2**_ **]** ^**3+**^ in its ^4^A_2_ ground state are collected in Table S1. Remarkably, the Cr–N_py_ and Cr–N_guanidine_ distances are very similar, in spite of the different donor strengths of pyridine and guanidine. The N–Cr–N angles are with 87–94° and 173–179° close to values for an ideal octahedron.

**Scheme 1:**
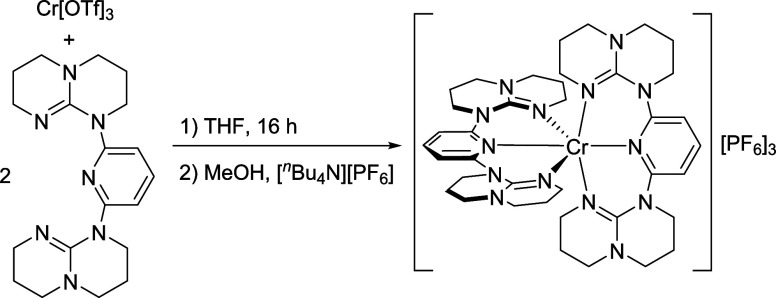
Synthesis of [Cr­(dgpy)_2_]­[PF_6_]_3_

The ATR IR spectrum of **[Cr­(dgpy)**_**2**_ **]­[PF**_**6**_ **]**_**3**_ **×1.5 DMF** crystals confirms the presence of DMF (ν̃_CO_ = 1670 cm^–1^) and PF_6_^–^ counterions (ν̃_PF_ = 829, 554 cm^–1^). The C–H vibrations of the ligand are observed at ν̃_CH,pyridine_ = 3119 cm^–1^ and ν̃_CH2,guanidine_ = 2960, 2876 cm^–1^ (Figure S4).^70^ The ESI^+^ mass spectrum of **[Cr­(dgpy)**_**2**_ **]­[PF**_**6**_ **]**_**3**_ in MeCN shows characteristic peaks for the trication **[Cr­(dgpy)**_**2**_ **]** ^**3+**^ (*m*/*z* = 253) and the ion clusters {**[Cr­(dgpy)**_**2**_ **]­[PF**_**6**_ **]**]}^2+^ (*m*/*z* = 452) and {**[Cr­(dgpy)**_**2**_ **]­[PF**_**6**_ **]**_**2**_]}^+^ (*m*/*z* = 1048) (Figure S5). In the cyclic voltammogram of **[Cr­(dgpy)**_**2**_ **]­[PF**_**6**_ **]**_**3**_ in [*n-*Bu_4_N]­[PF_6_]/MeCN, the complex cation **[Cr­(dgpy)**_**2**_ **]** ^**3+**^ is quasireversibly oxidized at *E*_p,ox_ = 1.68 V and irreversibly reduced at *E*_p,red_ = –1.56 V versus SCE (Figure S6a,b).

To further elucidate the instability after reduction in the condensed phase, we turned to gas phase studies in the mass spectrometer. Electron transfer dissociation (ETD) spectra were obtained from the **[Cr­(dgpy)**_**2**_ **]** ^**3+**^ and {**[Cr(dgpy)**_**2**_ **][PF**_**6**_ **]**}^2+^ species (Figures S7 and S8, Supporting Information). While the former is cleanly reduced to **[Cr­(dgpy)**_**2**_ **]** ^**2+**^ (*m*/*z* = 379) and **[Cr­(dgpy)**_**2**_ **]** ^**+**^ (*m*/*z* = 758), the ion cluster undergoes further reactions. After electron capture of {**[Cr(dgpy)**_**2**_ **][PF**_**6**_ **]**}^2+^ to give {**[Cr­(dgpy)**_**2**_ **]­[PF**_**6**_]}^+^ (*m*/*z* = 903), PF_5_ is cleaved off to yield {**[Cr­(dgpy)**_**2**_ **F]**}^+^ (*m*/*z* = 777) and finally dissociation of one dgpy ligand results in {**[Cr(dgpy)F]**}^+^ (*m*/*z* = 424). The fluorido ligands are presumably coordinated to the metal center in {**[Cr­(dgpy)**_**2**_ **F]**}^+^ and {**[Cr­(dgpy)­F**]}^+^ with a dgpy ligand partially and fully dissociated, respectively. This sequence is reasonably described as an intermediate associative substitution reaction^71^ at chromium after (metal-centered^72^) reduction. Analogous gas phase reactions were discussed for [Mn­(dgpy)_2_]^n+^ and PF_6_^–^ counterions.^73^ It is highly conceivable, that the reduced chromium­(III) complex undergoes similar substitution reactions with PF_6_^–^ nucleophiles in solution. Collision-induced dissociation (CID) mass spectrometric experiments confirm the higher lability of reduced species and contact ion pairs with the counterion (Figures S9 and S10). All chromium species show unspecific fragmentation patterns, which in part prevents assignments, and hints at fragmentation of the dgpy skeleton. If the counterion PF_6_^–^ is attached, the loss of PF_5_ is the dominant fragmentation channel. The CID breakdown curves suggest an increasing lability in the series **[Cr­(dgpy)**_**2**_ **]** ^**3+**^ < **[Cr­(dgpy)**_**2**_ **]** ^**2+**^ < {**[Cr­(dgpy)**_**2**_ **]­[PF**_**6**_ **]**}^2+^ < {**[Cr­(dgpy)**_**2**_ **]­[PF**_**6**_ **]**}^+^ ≈ {**[Cr­(dgpy)**_**2**_ **]­[PF**_**6**_ **]**_**2**_}^+^. In the condensed phase (MeCN solution), **[Cr­(dgpy)**_**2**_ **]** ^**3+**^ is stable in the presence of the comparably weakly coordinating anion PF_6_^–^, while **[Cr­(dgpy)**_**2**_ **]** ^**2+**^ appears unstable possibly reacting with the counterion (Figures S5–S10). To confirm the role of hexafluorophosphate in the irreversible reduction in solution, we turned to perchlorate as electrolyte in cyclic voltammetry experiments. Indeed, the reduction process becomes more reversible both in MeCN/[*n*-Bu_4_N]­[ClO_4_] and in DMF/[*n*-Bu_4_N]­[ClO_4_] with E12=−1.53V and −1.57 V, respectively (Figure S6c,d). Consequently, [*n*-Bu_4_N]­[PF_6_] is not the best choice as electrolyte for labile and highly charged redox intermediates.

### Optical Characterization of [Cr­(dgpy)_2_]­[PF_6_]_3_

UV/vis/NIR absorption, excitation and emission spectra of **[Cr(dgpy)**_**2**_ **][PF**_**6**_ **]**_**3**_ in MeCN are depicted in Figure 2. Spin-allowed charge transfer (^4^ILCT) and ligand field (^4^MC admixed with CT character) absorption bands are discernible below 400 nm and at 466 nm/520 (sh) nm, respectively, according to time-dependent density functional theory (TD-DFT) calculations and charge transfer number analysis (Table S2, Figure S11). The spin-forbidden NIR absorption band pattern between 680 and 850 nm (Figure 2) was fitted using five Voigt functions with maxima at 712, 740, 752, 781, and 809 nm (14045, 13515, 13300, 12800, and 12360 cm^–1^; Figure S12). The molar absorption coefficients ε are below 0.5 M^–1^ cm^–1^ due to the spin- and Laporte-forbidden^74^ nature of these transitions. CASSCF­(7,12)-NEVPT2 calculations on the ligandfield transitions at the FC geometry assign ^2^T_1_(3), ^2^E­(2), ^2^E­(1), ^2^T_1_(2), and ^2^T_1_(1) character to the spin- and Laporte-forbidden NIR absorption bands, respectively (Figures S13 and S14, Tables S3 and S4).

**Figure 2:**
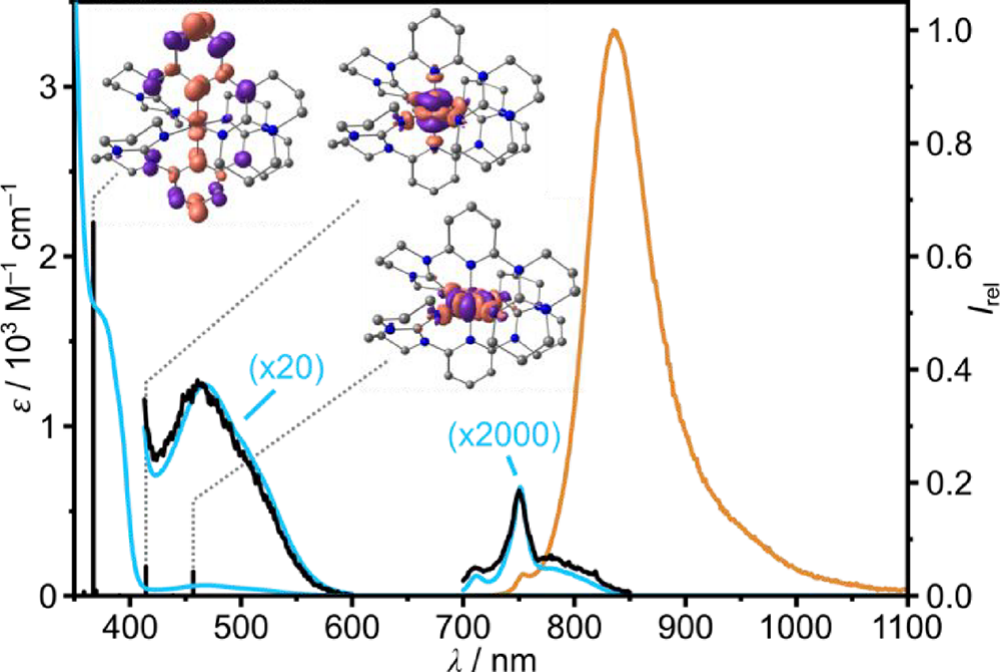
UV/vis/NIR absorption spectrum (blue), normalized excitation spectrum (*λ*_em_ = 836 and 875 nm for spin-allowed and spin-forbidden transitions, respectively, black) and normalized emission spectrum (*λ*_exc_ = 468 nm, orange) of **[Cr­(dgpy)**_**2**_ **]­[PF**_**6**_ **]**_**3**_ in deaerated MeCN at 293 K. The spin-forbidden and the low-energy spin-allowed absorption bands are scaled with the indicated factors. TD-DFT calculated oscillator strengths (black vertical bars) and difference electron densities of selected transitions.

Excitation at the ^4^MC absorption maximum throughout the visible spectral region and at the ^2^MC NIR bands (black excitation spectra in Figure 2) yields SF emission bands peaking at 754 nm (weak) and 835 nm (strong) with a full-width-at-half-maximum fwhm = 1010 cm^–1^ (orange emission spectrum in Figure 2). This energy is below that of typical emissive polypyridine chromium­(III) complexes, suggesting a stronger nephelauxetic effect^35–37^ of guanidine than pyridine donors (Figure 1).^10–17,75^ The stronger low-energy emission band possesses ^2^T_1_(1) → ^4^A_2_ character according to the CASSCF­(7,12)-NEVPT2 calculations (Table S4). However, the numerical agreement between experimental and calculated energies is somewhat poorer than typically observed for polypyridine chromium­(III) with this CASSCF method, even after scaling with an empirical scaling factor for the calculated SF energies^10,11^ (Figure S14). We attribute this to the effect of different donors, namely the four guanidines instead of pyridine donors. The experimental Stokes shift of 390 cm^–1^ (lowest absorption band at 809 nm, lowest emission band at 835 nm) is in the region typically observed for chromium­(III) SF emitters with rather nested excited doublet states.

The PL lifetime of *τ* = 63 μs at room temperature in MeCN is short (Figure S15) compared to that of benchmark molecular ruby complexes with millisecond lifetimes.^10–21^ Similarly, the PL quantum yield *Φ* = 0.5% is comparably low.^10–21^ The resulting radiative rate constant *k*_r_ = *Φ*/*τ* = 80 s^–1^, however, is in the typical range of spin- and Laporte forbidden transitions in chromium­(III) complexes.^2,10–21^ Hence, the low PL lifetime and quantum yield must be ascribed to efficient nonradiative decay pathways. Under air, the lifetime decreases marginally to 53 μs (Figure S15), suggesting only inefficient quenching of the doublet excited states by triplet oxygen.^13,34^ This inertness toward oxygen is based on the short excited state lifetime and the metal-centered nature of the excited state, hampering efficient wave function overlap for Dexter energy transfer.^13^

To elucidate thermally activated decay pathways such as bISC to ^4^MC states or thermally activated multiphonon relaxation, temperature-dependent PL studies were performed. The emission band develops a vibrational fine structure in frozen butyronitrile (*n*-PrCN) at 77 K with maxima centered at 816, 834, 863, and 925 nm (12,255; 11,990; 11,590; and 10,810 cm^–1^; fitted with Voigt functions, Figure S16). Progressions of similar magnitude have been observed for [Cr­(L^X^)_2_]^3+^ (X = O, S, CH_2_, NMe) and assigned to Cr–N vibrational modes.^10–17^ At 77 K, the PL lifetime increases by a factor of 5.7 to value of 360 μs (Figure S16) suggesting the presence of thermally activated decay pathways. However, millisecond lifetimes at 77 K as found for e.g. [Cr­(L^X^)_2_]^3+^ (X = O, S) are not achieved, suggesting efficient nonradiative decay even at 77 K.

To extract detailed kinetic information, temperature-dependent PL lifetime measurements were performed between 313 and 175 K in *n*-PrCN (Figure 3). This solvent remains liquid in this temperature range and rigidochromic effects are not expected. Two kinetic regimes can be estimated from an Arrhenius plot. The activation energies and pre-exponential factors amount to *E*_a1_ = 30 cm^–1^, *E*_a2_ = 2850 cm^–1^, and *A*_1_ = 5.8 × 10^3^ s^–1^, *A*_2_ = 2.7 × 10^10^ s^–1^, respectively. While the first process is essentially barrierless (small *E*_a1_) but inefficient (small *A*_1_), the second process is thermally activated (larger *E*_a2_) but more efficient (larger *A*_2_).

**Figure 3:**
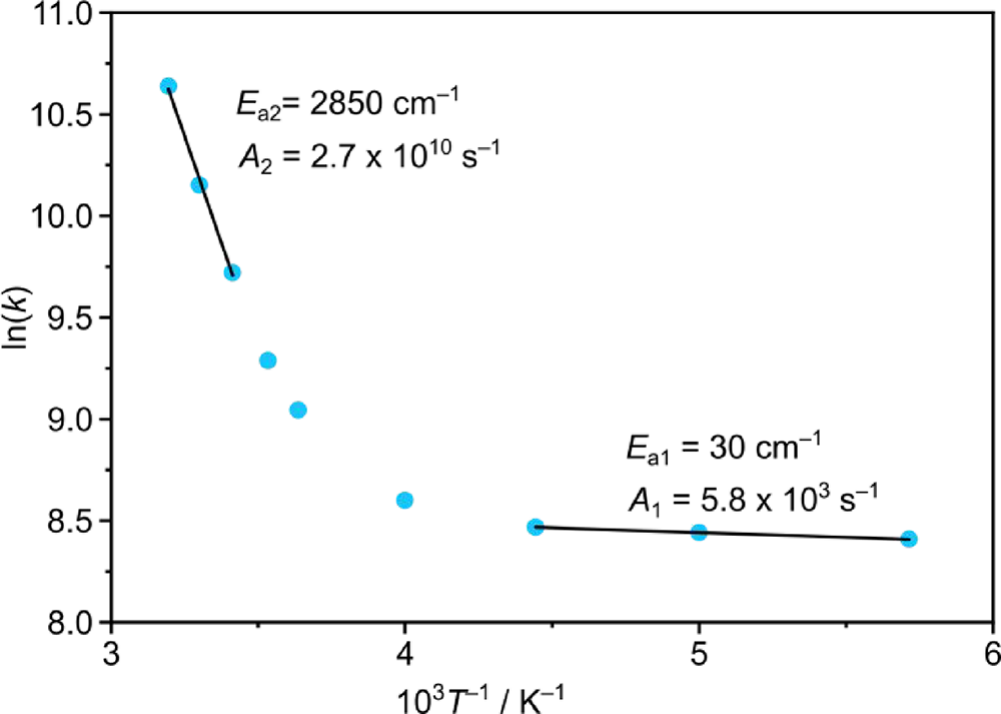
Arrhenius plot of the PL rate constants ln­(*k*(*T*)) vs *T* ^–1^ in the range *T* = 175–313 K (blue) with two linear fits (black) according to ln­(*k*) = ln­(*A*) + (−*E*_a_/(R*T*)) and *k*(*T*) = *τ*(*T*)^−1^. Measurements were performed in *n*-PrCN, which remains liquid in this temperature range.

Similarly, the complexes [Cr­(L^X^)_2_]^3+^ (X = O, S) show barriers of 370/3360 and 1090/4320 cm^–1^ with pre-exponential factors of 1.80 × 10^3^/9.18 × 10^9^ and 4.65 × 10^6^/6.27 × 10^14^ s^–1^, respectively.^10^ The larger barriers were associated with thermally activated bISC to distorted ^4^MC states, while the smaller ones were attributed to thermally activated energy transfer to C–H overtones by population of higher-energy SF states.^10^

In the present case, the barrier *E*_a2_ = 2850 cm^–1^ might be assigned to thermally activated bISC as well. Compared to the barriers of [Cr­(L^X^)_2_]^3+^ (X = O, S), this barrier is lower by 510 and 1470 cm^–1^, respectively. Hence, in **[Cr­(dgpy)**_**2**_ **]** ^**3+**^, bISC dominates the decay to a larger extent. A strongly distorted metal-centered state could be localized by TD-DFT geometry optimization (Table S1). Indeed, this geometry optimized ^4^MC state has elongated Cr–N_py_ bonds up to 2.34 Å, a reduced intraligand N_gua_–Cr–N_guanidine_ angle of 157° and a significant tilting of the pyridines with Cr–N_py_–C_para_ of 160° as compared to Cr–N_py_ = 2.04 Å, N_guanidine_–Cr–N_guanidine_ = 173° and Cr–N_py_–C_para_ = 179° in the ^4^A_2_ ground state. This optimized ^4^MC state at 2.07 eV is lower by 0.64 eV than the respective FC state (*E*_DFT,FC_ = 2.71 eV) suggesting a major energy gain upon distortion. The energy lowering of the ^4^MC state might be sufficient to enable thermally activated bISC from the ^2^MC state(s) to the distorted ^4^MC state(s). Furthermore, the large frequency factor *A*_2_ = 2.7 × 10^10^ s^–1^ suggests sufficient coupling of the doublet and quartet excited states.

To probe the second, nearly barrierless process, we focus on C–H oscillators of the dgpy ligand that are close to the metal center, i.e., guanidine ring-methylene units, which could contribute to the nonradiative excited state decay (*d* ^–6^ dependency, eq 2). The shortest Cr**^...^** CH_2_ distances amount to *d*_1_ = 3.0 Å with the equatorial hydrogen atom of the CH_2_ unit being closer to the metal than the axial one. The other five CH_2_ groups possess Cr**^...^** CH_2_ distances of *d*_*n*_ = 4.4, 4.9, 5.4, 5.4, and 4.7 Å (*n* = 2–6) and these will contribute less to *k*_nr_ (eq 2).

While overtone spectra of ring CH_2_ groups, e.g. cyclohexane, have been reported (ν_CH_^2,3,4,5^ ≈ 5700, 8300, 10800, and 13100 cm^–1^),^67^ data for bicyclic guanidines are lacking. We chose the soluble 1,5,7-triazabicyclo[4.4.0]­dec-5-ene as ligand surrogate without interfering C–H modes of pyridine (Figure S17).^76^ To eliminate the contribution of the N–H overtones of 1,5,7-triazabicyclo[4.4.0]­dec-5-ene, we also recorded the IR overtone spectrum of the *N*-deuterated derivative^77^ in the spectral region of the **[Cr­(dgpy)**_**2**_ **]** ^**3+**^ emission band (Figures 4a and S18). In fact, significant spectral overlap exists between the SF luminescence band of **[Cr­(dgpy)**_**2**_ **]** ^**3+**^ and the ν_CH_^4^ band of the *N*-deuterated model guanidine at 293 K (Figure 4a). Additionally, small spectral overlap of the weak higher energy emission is seen with the ν_CH_^5^ band around 13,000 cm^–1^. A combination band below 10,000 cm^–1^ also contributes to the spectral overlap (Figure 4a).

**Figure 4:**
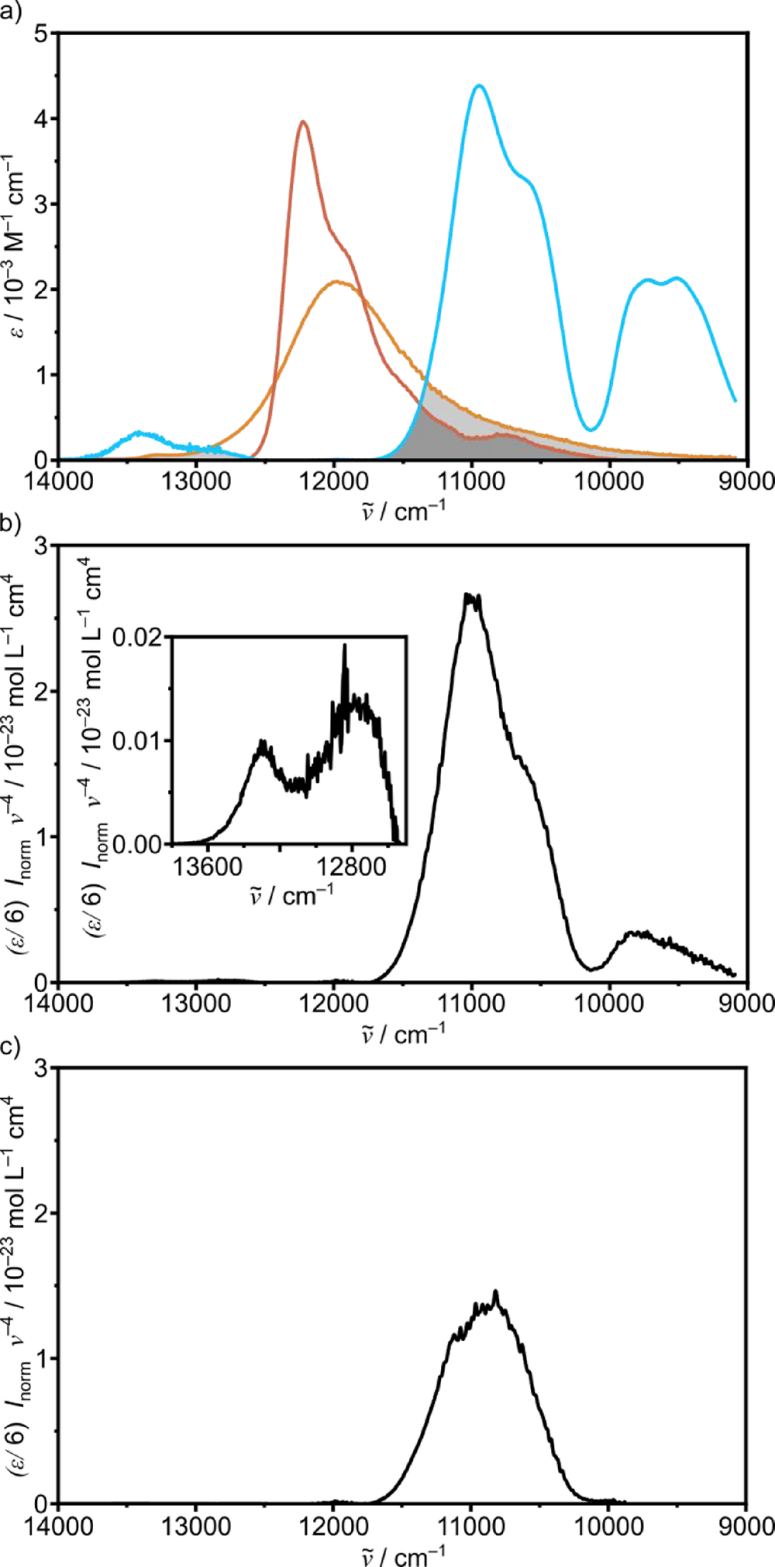
(a) Emission spectra of **[Cr­(dgpy)**_**2**_ **]­[PF**_**6**_ **]**_**3**_ at 293 K (orange, in MeCN) and 77 K (red, in *n*-PrCN) and NIR absorption spectrum of *N*-deuterated 1,5,7-triazabicyclo[4.4.0]­dec-5-ene (blue) in CD_2_Cl_2_. Plots of the product of the extinction coefficients of *N*-deuterated 1,5,7-triazabicyclo[4.4.0]­dec-5-ene (normalized to one CH_2_ unit) in CD_2_Cl_2_ and the emission intensity (normalized to a unit area) of **[Cr­(dgpy)**_**2**_ **]** ^**3+**^, scaled with ν̃^–4^ in the overtone regions of ν_CH_^4^ and ν_CH_^5^ in MeCN (b) at 293 K and (c) at 77 K (eq 3). The inset in (b) shows a close-up of the ν_CH_^5^ overtone region.

As a measure of the SOI that is responsible for the energy transfer to individual CH_2_ overtones, we plotted the product of the area-normalized emission spectrum of **[Cr­(dgpy)**_**2**_ **]­[PF**_**6**_ **]**_**3**_ at 293 K and the NIR overtone spectrum of *N*-deuterated 1,5,7-triazabicyclo[4.4.0]­dec-5-ene (divided by 6 to account for the six CH_2_ units of the bicyclic model) scaled with ν̃^–4^ (eq 3, Figure 4b). Several C–H overtone regions contribute to the total SOI, namely the third and fourth C–H overtones ν_CH_^4^ and ν_CH_^5^ at around 11,000 and 13,400 cm^–1^, respectively, in addition to a combination band at around 9800 cm^–1^. At 77 K, however, the overlap between ν_CH_^5^ and the area-normalized emission vanishes due to depopulation of the higher SF states (Figure 4a). More importantly, the emission band sharpens at 77 K (fwhm = 540 cm^–1^), which leads to a significantly smaller spectral overlap in the ν_CH_^4^ and combination mode regions (Figure 4c). The calculated SOIs for a single CH_2_ moiety amount to 2.1 × 10^–20^ and 1.2 × 10^–20^ M^–1^ cm^3^ at 293 and 77 K, respectively. The nonradiative rate constant *k*_nr_ of **[Cr­(dgpy)**_**2**_ **]** ^**3+**^ (four guanidines with six CH_2_ units each) scales with the sum of the *d* ^–6^ weighted SOI of a single CH_2_ unit (eq 2). Weighted summation over all six CH_2_ units per guanidine with the respective Cr**^...^** CH_2_ distances multiplied by four to account for the four guanidines of the two dgpy ligands in **[Cr­(dgpy)**_**2**_ **]** ^**3+**^ gives a factor $f=4\sum_{n=1}^{n=6}{d_{n}}^{-6}=0.007$ with the closest CH_2_ unit at a distance *d*_1_ contributing 78% to *f*. Irrespective of inclusion of only the closest (*d*_1_) or all CH_2_ units (4 × (*d*_1_ – *d*_6_)), the SOI increases by a factor of 1.75 at 293 K as compared to 77 K and hence *k*_nr_ should increase by a factor of 1.75 as well, based on the resonant energy transfer relaxation path. As the radiative rate constant *k*_r_ = *Φ*/*τ* = 80 s^–1^ should be temperature-independent,^78–80^ we can estimate *k*_nr_(293 K) = 15,800 s^–1^ and *k*_nr_(77 K) = 2700 s^–1^. The experimental ratio of *k*_nr_(293 K)/*k*_nr_(77 K) = 5.85 is larger than the estimated increase via multiphonon relaxation only (factor of 1.75). The remaining contribution to excited state relaxation at increasing temperature hence stems from thermally activated bISC to ^4^MC states. In the present complex, bISC is the dominant decay path at higher temperatures, while resonant energy transfer dominates at lower temperature (Figure 3).

The alternative assignment of the larger barrier *E*_a2_ to thermally activated resonant energy transfer to the higher-energy ν_CH_^5^ overtone (12,600–13,500 cm^–1^) by thermal population of higher-energy SF states (^2^T_1_(2), ^2^E­(1), ^2^E­(2) states at 12,809, 13,306, 13,516 cm^–1^) seems possible as well based on thermodynamics. However, the drastically different pre-exponential factors *A*_1_ and *A*_2_ (Figure 3) for both processes suggest different mechanisms for the two processes. This is further supported by the similar behavior of [Cr­(L^X^)_2_]^3+^ (X = O, S) complexes.^10^

## Summary and Conclusions

This study details the thermally activated and nonactivated relaxation pathways of the emissive doublet states in a chromium­(III) complex **[Cr­(dgpy)**_**2**_ **]** ^**3+**^ coordinated with bicyclic guanidine and pyridine donor units (dgpy = 2,6-diguanidylpyridine). The guanidine donor shifts the spin-flip emission energy bathochromically as compared to pyridine donors (nephelauxetic effect). The radiative rate constant is small with *k*_r_ = 80 s^–1^ due to the spin- and Laporte-forbidden nature of the transition, typical for chromium­(III) complexes. On the other hand, the nonradiative rate constant is with *k*_nr_ = 15800 s^–1^ at 293 K quite large for molecular rubies. Two temperature-dependent processes contribute to *k*_nr_, namely thermally activated back-intersystem crossing to distorted metal-centered quartet states with elongated Cr–N_py_ bonds with an activation barrier of 2850 cm^–1^ and resonant energy transfer to the third and fourth vibrational overtones ν_CH_^4/5^ and overtone combination bands of the CH_2_ units. This energy transfer is a temperature-dependent process as well, due to spectral narrowing of the emission band at 77 K to 53% of the original fwhm, which diminishes the spectral overlap with ν_CH_^4^ and combination modes, and depopulation of higher-energy doublet states, which reduces the spectral overlap with the ν_CH_^5^ overtone at lower temperature.

The dgpy ligand provides two nonradiative relaxation pathways, first by distortion of the coordination sphere with efficient back-intersystem crossing, which is facilitated by the flexible guanidine/pyridine units, and second by methylene oscillators close to the chromium­(III) center, which enable resonant energy transfer. As the third C–H overtone and combination bands span a spectral region from 11,500 to 9000 cm^–1^ (870–1110 nm), chromium­(III) complexes with CH_2_ units close to the metal center are not expected to strongly emit in this NIR spectral region (Figure 1b) and hence aliphatic units within the ligand coordination should be avoided when luminescence in this spectral region is targeted.

## Experimental Section

### General Procedures

The ligand dgpy^69^ and Cr­[OTf]_3_ ^10^ were synthesized according to literature procedures. 1,5,7-Triazabicyclo[4.4.0]­dec-5-ene was *N*-deuterated according to a literature procedure.^77^ All reagents were used as received from commercial suppliers (abcr, Acros Organics, Alfa Aesar, Thermo Fisher Scientific, Sigma-Aldrich and TCI) if not noted otherwise. Solvents were dried by refluxing over potassium (THF, toluene, C_6_D_6_), sodium (diethyl ether) or calcium hydride (MeCN, CD_2_Cl_2_) and distillation. Butyronitrile *n*-PrCN was purified according to a literature procedure.^81^ [*n*-Bu_4_N]­[PF_6_] for electrochemical experiments (≥99% for electrochemical analysis, Sigma-Aldrich) was dried at 80 °C and reduced pressure (10^–3^ mbar) for 3 days and stored under argon. Syntheses and handling of air-sensitive compounds were either conducted using Schlenk techniques or a glovebox (UniLab/Mbraun – Ar 5.0; O_2_ < 0.1 ppm; H_2_O < 0.1 ppm).

**NMR spectra** were recorded on a Bruker Avance II 400 spectrometer at 400.13 MHz (^1^H). Proton resonances are reported in ppm versus the solvent signal as an internal standard *δ*(benzene) = 7.16 ppm.^82^ (s) = singlet, (d) = doublet, (t) = triplet, (m) = multiplet.

**IR spectra** were recorded with a Bruker Alpha II FTIR spectrometer with an ATR unit containing a diamond crystal. The intensities were qualitatively indicated with weak (w), medium (m) and strong (s).

**ESI** ^**+**^ **mass spectra** were measured on an Agilent 6545 HPLC-ESI-QTOF-MS spectrometer in MeCN.

**ESI** ^**+**^ **mass spectra for ETD and CID experiments** were recorded using a Bruker amZon ETD 3D Paul trap mass spectrometer, which is operated at room temperature. The sample solution of **[Cr(dgpy)**_**2**_ **][PF**_**6**_ **]**_**3**_ was prepared in dry and degassed MeCN with concentrations of 10^–4^–10^–5^ M. The sample solution was continuously infused into the spray chamber using a syringe pump. Nitrogen was used as nebulizer gas and dry gas. The mass spectrometer is equipped with a negative chemical ionization (nCI) module to allow for electron transfer dissociation/reduction (ETD/ETR) experiments. Fluoranthene radical anions are prepared via chemical ionization. These are transferred into the ion trap where they can interact/react with the stored ions. For ETD experiments, the target ion was isolated and stored in the ion trap for 300 ms to allow for enough time for the electron transfer reaction. Collision induced dissociation (CID) is used to induce fragmentation in a chosen precursor ion. Ions are stored in the 3D Paul trap (ion trap) of the mass spectrometer by a combination of DC voltage and radio frequency (RF). The trapped ions are accelerated by increasing the RF amplitude. Repeated collisions with the helium buffer gas transfer kinetic energy from the collisions into rotational–vibrational degrees of freedom. Internal vibrational redistribution leads to heating up of the stored ion and subsequently to fragmentation of the weakest bond.^83,84^ The full isotopic pattern of the respective chromium species was isolated prior to CID experiments. CID breakdown curves are recorded by stepwise increase of the excitation amplitude (*E*_LAB_) from 0 to 3 V in 0.02 V steps. At each excitation amplitude, mass spectra were recorded for 300 ms. At each step mass spectra were recorded for a total of 18 s and averaged afterward. Relative signal intensities are calculated according to eq 4, with *I*_abs_^*P*^ being the absolute intensity of the respective parent ion, and *I*_abs_^*F*_*i*_^ the absolute fragment intensities.

$$I_{\mathrm{rel}}^{P}(E_{\mathrm{LAB}})=\frac{\sum_{i}I_{\mathrm{abs}}^{P_{i}}(E_{\mathrm{LAB}})}{\sum_{i}I_{\mathrm{abs}}^{F_{i}}(E_{\mathrm{LAB}})+\sum_{i}I_{\mathrm{abs}}^{P_{i}}(E_{\mathrm{LAB}})}$$ 4

The instrument specific voltage applied as excitation amplitude, i.e., *E*_LAB_, was corrected in two steps to consider the different masses and different charge states *z* of the investigated ions.

$$E_{\mathrm{COM}}=E_{\mathrm{LAB}}\times \frac{m_{\mathrm{He}}}{m_{\mathrm{He}}+m_{\mathrm{ion}}}$$ 5

$$E_{\mathrm{COMz}}=E_{\mathrm{COM}}\times z$$ 6

The obtained intensities were fitted with a sigmoidal function. The obtained *E*_COMz_^50%^ value is where half of the parent ions are fragmented and it can be used as a qualitative measure for the relative stabilities of ions of chemically similar structures.^85^

$$I_{\mathrm{fit}}^{P}(E_{\mathrm{COMz}})=\frac{A}{1+e^{-B(E_{\mathrm{COMz}-}E_{\mathrm{COMz}}^{50\%})}}$$ 7

The parameter *A* describes the fitted intensity of the parent ion and the exponent the slope of the sigmoidal curve.

**Electrochemical experiments** were carried out on a BioLogic SP-200 voltammetric analyzer. The measurements were performed in a glovebox, using dry MeCN or dry DMF as the solvent. The working and counter electrodes consisted of platinum wire and 10 mM Ag/AgNO_3_ (100 mM [*n*-Bu_4_N]­[ClO_4_] in acetonitrile) was used as the reference electrode. 100 mM [*n*-Bu_4_N]­[PF_6_] or [*n*-Bu_4_N]­[ClO_4_] as supporting electrolytes and 1 mM of the sample were used. Cyclic voltammetry experiments were carried out at scan rates of 50–100 mV s^–1^. Potentials were referenced relative to the ferrocenium/ferrocene couple. Potentials were converted to the saturated calomel electrode (SCE) according to the literature.^86^

The **elemental analysis** was conducted by the central analytic service of the Department of Chemistry of the Johannes Gutenberg University Mainz using an Elementar vario EL Cube.

**UV/vis/NIR absorption spectra** were recorded with an Agilent Cary 5000 UV/vis/NIR spectrophotometer, using 1.00 cm quartz cells. When needed, 1.00 cm quartz cells with a Schott valve were used to maintain an inert atmosphere. For the measurement of the spin-forbidden absorption bands, a cuvette with a path length of 10.0 cm was used. The spin-forbidden NIR absorption bands were baseline corrected using a biexponential function (eq 8) to model the tail of the spin-allowed absorption bands. Deconvolution of the baseline corrected spectrum was achieved with five Voigt functions using OriginPro 2024.

$$\varepsilon (\tilde{v})=\varepsilon_{0}+A_{1}e^{\tilde{v}-{\tilde{v}}_{0}/t_{1}}+A_{2}e^{\tilde{v}-{\tilde{v}}_{0}/t_{2}}$$ 8

**Emission spectra** and **luminescence decay curves** were recorded with a FLS1000 spectrometer from Edinburgh Instruments equipped with the cooled red and NIR sensitive photomultiplier detectors PMT-980 and N-G09 PMT-1700, together covering the entire spectral range between 200 and 1700 nm. A xenon arc lamp Xe2 (450 W) was used for excitation in steady-state measurements. Time-resolved luminescence experiments were performed in the multichannel scaling mode employing a variable pulsed laser VPL-450 (*λ*_exc_ = 451.3 nm) as excitation source. The absolute luminescence quantum yield *Φ* was determined using an integrating sphere from Edinburgh Instruments. Relative uncertainty of *Φ* is estimated to be ±10%. Room temperature measurements were conducted in MeCN (Optima LC/MS grade, Fisher Scientific). Low temperature measurements were conducted in purified butyronitrile *n*-PrCN using a liquid nitrogen cooled cryostat Optistat DN from Oxford Instruments. Deconvolution of the emission spectrum at 77 K was achieved with four Voigt functions using OriginPro 2024.

**Intensity data for crystal structure determination of [Cr(dgpy)**_**2**_ **][PF**_**6**_ **]**_**3**_ were collected with a STOE IPDS-2T diffractometer from STOE & CIE GmbH with an Oxford cooling using Mo–K_α_ radiation (*λ* = 0.71073 Å). The diffraction frames were integrated using the STOE X-Area^87^ software package and were corrected for absorption with MULABS^88^ of the PLATON software package.^89^ The structures were solved with SHELXT^90^ and refined by the full-matrix method based on *F* ^2^ using SHELXL^91^ of the SHELX^92^ software package and the ShelXle^93^ graphical interface. All non-hydrogen atoms were refined anisotropically while the positions of all hydrogen atoms were generated with appropriate geometric constraints and allowed to ride on their respective parent atoms with fixed isotropic thermal parameters. Crystallographic data for the structures reported in this paper have been deposited with the Cambridge Crystallographic Data Centre as supplementary publication no. CCDC-2448606.

### Crystallographic Data of [Cr­(dgpy)_2_]­[PF_6_]_3_×1.5DMF

C_42.50_H_64.50_CrF_18_N_15.50_O_1.50_P_3_ (1303.50); monoclinic; *Ia, a* = 35.485(7) Å, *b* = 17.866(4) Å, *c* = 16.787(3) Å, β = 94.21(3)°; *V* = 10614(4) Å^3^, *Z* = 8; density (calculated) = 1.631 g cm^–3^; *T* = 120(2) K; μ = 0.423 mm^–1^; *F*(000) = 5368; crystal size 0.400 × 0.280 × 0.090 mm^3^; θ = 2.433–28.245 deg; −47 ≤ *h* ≤ 46, −23 ≤ *k* ≤ 23, −18 ≤ *l* ≤ 22; rfln collected = 75823; rfln unique = 23321 [*R*(int) = 0.0968]; completeness to θ = 25.242 deg. = 99.9%; semi empirical absorption correction from equivalents; max. and min transmission 1.4293 and 0.6544; Data 23321; restraints 3430; parameters 1815; goodness-of-fit on *F* ^2^ = 1.617; final indices [*I* > 2σ­(*I*)] *R*_1_ = 0.1230, *wR*_2_ = 0.3515; *R* indices (all data) *R*_1_ = 0.1431, *wR*_2_ = 0.3775; largest diff. peak and hole 3.142 and −1.018 e Å^–3^.

**Density functional theory (DFT) and CASSCF-NEVPT2 calculations** were carried out using the ORCA program package 5.0.3.^94,95^

**DFT** calculations were performed using the B3LYP functional^96,97^ employing the RIJCOSX approximation^98,99^ and the SARC/J auxiliary basis.^100^ Tight convergence criteria were chosen for DFT-UKS calculations (keywords *tightscf* and *tightopt*). Relativistic effects were calculated at the zeroth order regular approximation (keyword *ZORA*) level.^100^ The ZORA keyword automatically invokes relativistically adjusted basis sets. To account for solvent effects, a conductor-like screening model (keyword *CPCM*) modeling MeCN was used in all calculations.^101,102^ Geometry optimizations were performed using Ahlrichs’ polarized valence triple-ζ basis set (def2-TZVPP).^103,104^ Atom-pairwise dispersion correction was performed with the Becke-Johnson damping scheme (keyword *D3BJ*).^105,106^ The energy of the electronic ground states (quartet and doublet) and the presence of energy minima were checked by numerical frequency calculations. Explicit counterions and/or solvent molecules were not taken into account. TD-DFT calculations were performed at the same level of theory. Fifty vertical spin-allowed transitions were calculated. The assignment of the state characters has been done dividing the molecule into three fragments (metal center, two pyridine and two bis­(guanidine) moieties) and calculating charge transfer (CT) numbers, as implemented in the TheoDore software package.^107,108^ Excited state geometry optimization of the ^2^MC state was done using DFT and setting the multiplicity to 2. Exited state geometry optimization of the lowest energy ^4^MC state was done using TD-DFT and the given state was selected according to the TD-DFT calculation at the initial geometry (keyword *iroot*). To prevent root flipping after a step during the optimization, the total overlap between the excited state wave functions was calculated and compared with the previous one (keyword *followiroot*).

**CASSCF­(7,12)-SC-NEVPT2** calculations of ground and excited state properties with respect to metal-centered (MC) states were performed using the complete-active-space self-consistent field method (CASSCF).^109,110^ To accelerate the calculation, the RI-JK approximation was used,^111,112^ with an automatically generated auxiliary basis (keyword *AutoAux*). To recover the missing dynamic electron correlation, the strongly contracted variant of N-electron valence perturbation theory to second order (SC-NEVPT2) was used.^113,114^ All electronic states are classified by irreducible representations of the *O* point group, despite the lower actual symmetry of the considered complex. To accurately model the ligand field, the active space was expanded to encompass the dominant bonding/antibonding orbitals formed between chromium and the ligand. In addition to the minimal active space of (3,5), two occupied Cr–N σ bonding orbitals and a second d shell^115^ were included in these calculations giving an active space of (7,12). Ten quartet and 10 doublet roots were calculated with this active space.

### Synthesis of dgpy

The ligand dgpy was synthesized according to a modified literature procedure from 2,6-dibromopyridine and 1,3,4,6,7,8-hexahydro-2H-pyrimido­[1,2-*a*]­pyrimidine using KO*^t^* Bu and Pd­(OAc)_2_/BINAP in toluene,^69^ using Schlenk techniques and dry solvents to avoid protonation. The product dgpy was extracted from the beige crude product containing KBr with warm toluene (5 × 50 mL, 50 °C). The solvent was removed from the combined extracts under reduced pressure. The remaining beige solid was washed with dry diethyl ether (2 × 25 mL) and dried under reduced pressure to yield a beige powder. ^1^H NMR (400 MHz, C_6_D_6_): *δ*/ppm = 7.91 (d, ^3^ *J* = 8.0 Hz, 2H), 7.36 (t, ^3^ *J* = 8.0 Hz, 1H), 3.83–3.80 (m, 4H), 3.53 (t, ^3^ *J* = 5.7 Hz, 4H), 2.74 (t, ^3^ *J* = 6.0 Hz, 4H), 2.58 (t, ^3^ *J* = 6.5 Hz, 4H), 1.63–1.52 (m, 8H).

### Synthesis of [Cr­(dgpy)_2_]­[PF_6_]_3_

To avoid protonation of the strongly basic dgpy ligand under ambient conditions, which would hamper the complexation of chromium­(III), the complex synthesis was conducted in a glovebox. Cr­[OTf]_3_ ^10^ (141 mg, 0.28 mmol, 1.0 equiv) was dissolved in dry THF (20 mL). This solution was added dropwise to a solution of dgpy (200 mg, 0.57 mmol, 2.0 equiv) in dry THF (5 mL) yielding an orange-brown precipitate. The mixture was stirred for 16 h at 298 K. The reaction vessel was removed from the glovebox. The supernatant was decanted, the remaining solid was washed with THF (5 × 5 mL) and diethyl ether (5 mL) and dried under air. The solid was dissolved in methanol (5 mL), yielding an orange solution. The complex solution was added dropwise to a solution of [*n-*Bu_4_N]­[PF_6_] (542 mg, 1.4 mmol, 5.0 equiv) in methanol (10 mL) yielding an orange to brown precipitate. The supernatant was decanted, washed with diethyl ether (5 mL) and dried under air. To ensure quantitative salt metathesis, the complex was dissolved in 5 mL of acetonitrile, containing [*n*-Bu_4_N]­[PF_6_] (542 mg, 1.4 mmol, 5.0 equiv) and crystallized via diethyl ether diffusion. The resulting orange crystals were washed with methanol (5 × 5 mL) and diethyl ether (5 mL), dried and recrystallized from DMF/diethyl ether, yielding large orange crystals (140 mg, 0.12 mmol; 43% yield). The sample used for elemental analysis was recrystallized from MeCN/Et_2_O. The isolated crystals were pulverized and dried under reduced pressure for 3 days. Elemental analysis: calcd (%) for C_38_H_54_CrF_18_N_14_P_3_ (1193.84 g/mol): C, 38.23; H, 4.56; N, 16.43; found: C, 38.47; H, 4.85; N, 16.50. MS (MeCN/ESI^+^): calcd for {[Cr­(dgpy)_2_]­[PF_6_]_2_}^+^: *m*/*z* = 1048.334, found: *m*/*z* (%) = 1048.333 (28.6); calcd for {[Cr­(dgpy)_2_]­[PF_6_]}^2+^: *m*/*z* = 451.684, found: *m*/*z* (%) = 451.684 (100); calcd for [Cr­(dgpy)_2_]^3+^: *m*/*z* = 252.801, found: *m*/*z* (%) = 252.801 (83.52). IR (ATR): ν̃/cm^–1^ = 3119 (w, CH), 2960 (w, CH), 2876 (w, CH), 1670 (w, C=O (DMF crystal solvate)), 1594 (s), 1504 (m), 1460 (m), 1432 (m) 1381 (m), 1328 (m), 1309 (m), 1208 (m), 1161 (m), 1070 (w), 1044 (w), 1028 (m), 877 (w), 829 (vs, PF), 754 (m), 719 (m), 554 (vs, PF), 427 (w). UV/vis/NIR (MeCN): *λ*/nm (*ε*/M^–1^ cm^–1^): 324 (10100), 380 (1200, sh), 393 (750, sh), 466 (60), 520 (40, sh), 712 (0.1), 751 (0.3), 800 (0.1, sh). Emission (MeCN, 293 K): *λ*/nm = 754, 835. Emission Lifetime (MeCN, 293 K): *τ*/μs = 63 (deaerated); 53 (aerated). Emission (*n*-PrCN, 77 K): *λ*/nm = 816, 834, 863, 925. Quantum Yield (MeCN, 293 K): *Φ*/% = 0.5. CV (MeCN, 100 mM [*n*-Bu_4_N]­[PF_6_]): *E*_p, red_/V vs SCE = −1.56, *E*_p, ox_/V vs SCE = 1.68.

## Supplementary Material





## Notes

Analytical and spectroscopic data, results of quantum chemical calculations (PDF)
Cartesian coordinates of optimized geometries from quantum-chemical calculations (XYZ)
