# Supplementary material for: Thermally Activated and Nonactivated Excited State Decay of [Cr(dgpy)2]3+
Source: Inorg Chem. 2025 Oct 1;64(40):20404–14. doi: 10.1021/acs.inorgchem.5c03668 (PMC12522133; doi:10.1021/acs.inorgchem.5c03668)
Supplement: Supplementary file 1 [file ic5c03668_si_001.pdf]

Supporting Information

Thermally Activated and Non-activated Excited State Decay of $[Cr(dgpy)_2]^{3+}$

Steven Sittel,[a] Robert Naumann,[a] Christoph Förster,[a] Maximilian E. Huber,[b] Jennifer Meyer,[b] and Katja Heinze[a]*

[a] Department of Chemistry, Johannes Gutenberg University Mainz, Duesbergweg 10-14, 55128 Mainz (Germany), email katja.heinze@uni-mainz.de

[b] Fachbereich Chemie und Forschungszentrum OPTIMAS, RPTU Kaiserslautern-Landau, Erwin-Schroedinger Str. 52, 67663 Kaiserslautern (Germany)

Chart S1. a) Pseudo-octahedral chromium(III) complexes with strong-field ligands, but highly variable PL quantum yields.

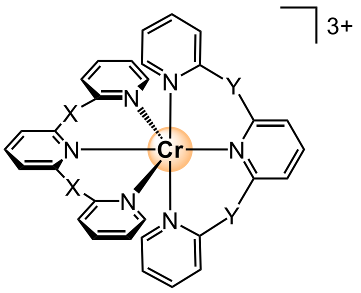

$[Cr(L^X)(L^Y)]^{3+}$
(X, Y = NMe, CH_2, O, S)

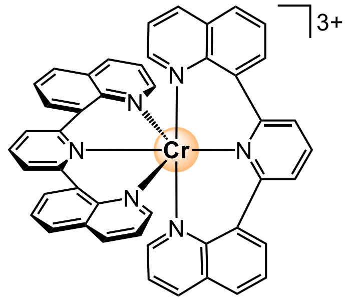

$[Cr(dqp)_2]^{3+}$

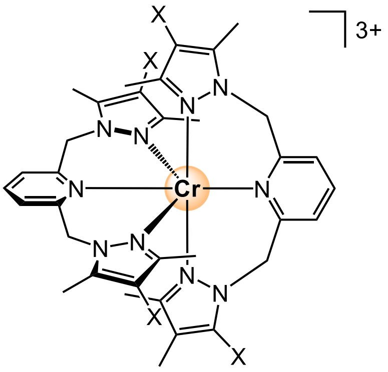

$Cr(XMebipzp)_2]^{3+}$
(X = H, I)

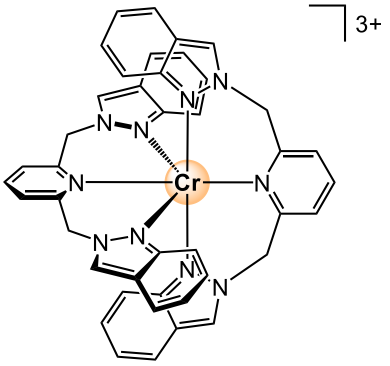

$Cr(bip^*)_2]^{3+}$

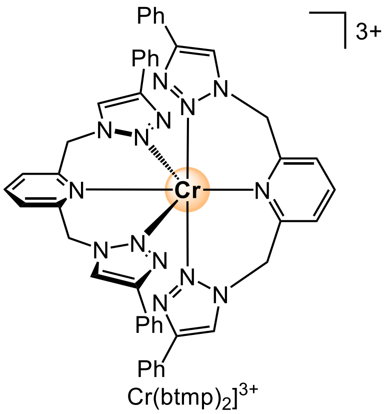

$Cr(btmp)_2]^{3+}$

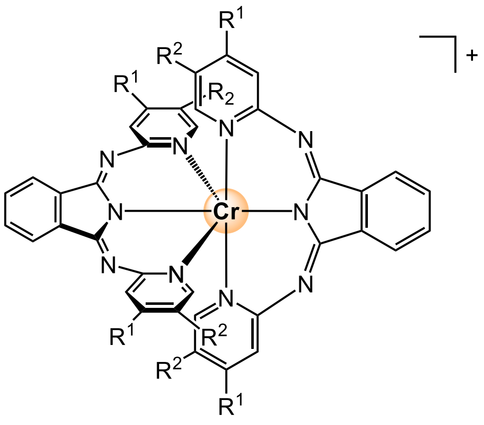

$[Cr(bpi^{R1R2})_2]^{+}$
($R^{1,}$ R^2 = H, Me, OMe, NMe_2)

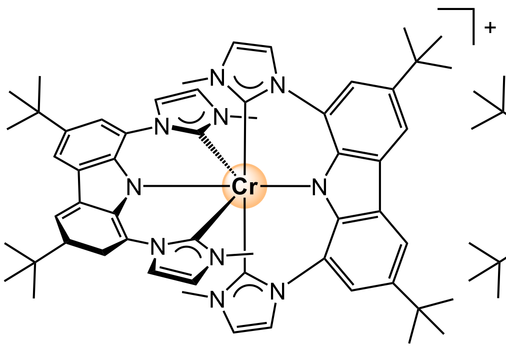

$[Cr(bimca)_2]^{+}$

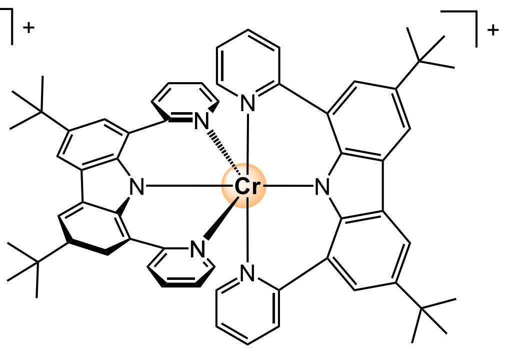

$[Cr(dpc)_2]^{+}$

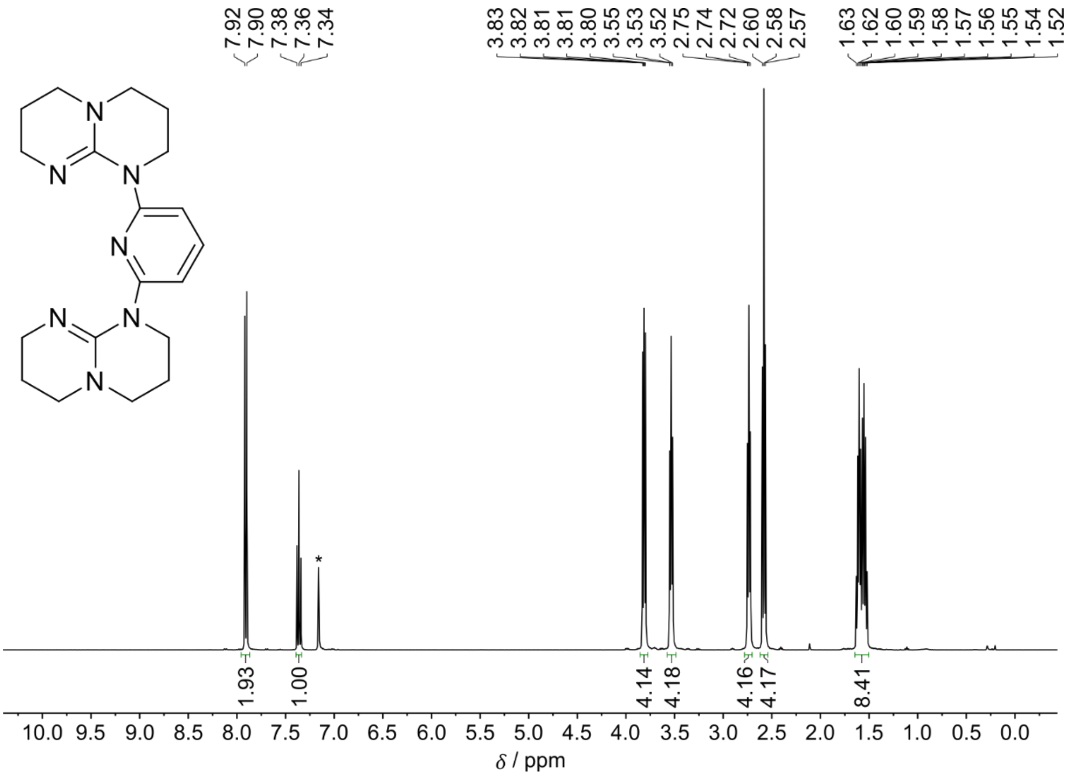

Figure S1. ^{1}H NMR spectrum of dried dgpy in C_6D_6. The asterisk * denotes benzene solvent resonance. The spectrum confirms the absence of water and guanidine protonation.

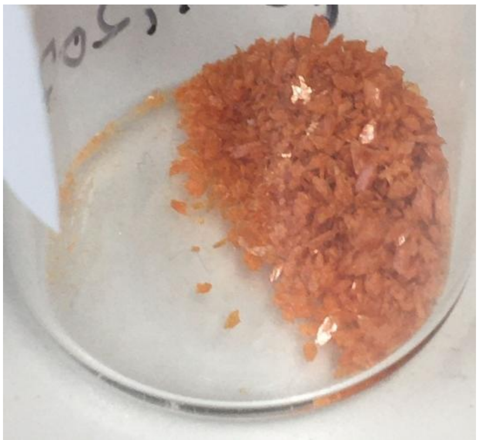

Figure S2. Photograph of crystals of **[Cr(dgpy)$_2$][PF$_6$]$_3$**$\times$1.5 DMF.

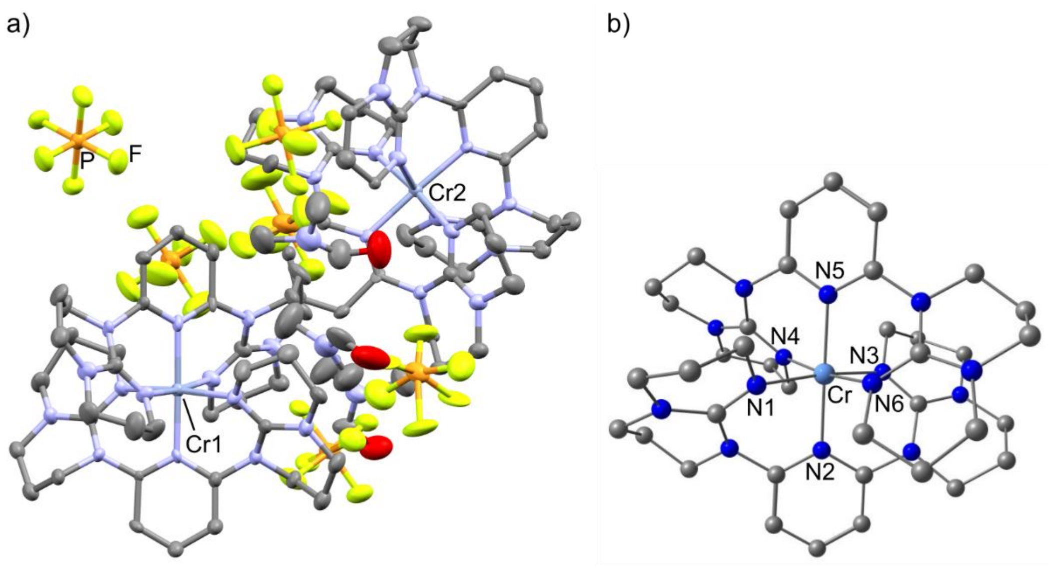

Figure S3. a) Molecular structure of **[Cr(dgpy)$_2$][PF$_6$]$_3$**×1.5 DMF with two independent cations in the unit cell. Thermal ellipsoids set to 50 % probability. b) DFT-calculated structure of **[Cr(dgpy)$_2$]$^{3+}$**. The numbering of atoms corresponds to that used in Table S1. Hydrogen atoms were removed for clarity.

Table S1. Bond lengths [Å] and angles [deg] of **[Cr(dgpy)$_2$]$^{3+}$** obtained from DFT calculations in the optimized $^4A_2$ ground state, the optimized lowest-energy ^{2}MC state and the optimized lowest-energy ^{4}MC state ($^4T_2(1)$).

	**$^4A_2$**	**^{2}MC**	**^{4}MC**
Cr–N1	2.040	2.035	2.054
Cr–N2	2.045	2.039	2.177
Cr–N3	2.041	2.031	2.036
Cr–N4	2.042	2.042	2.107
Cr–N5	2.042	2.038	2.341
Cr–N6	2.038	2.019	1.976
N1–Cr–N2	87.4	87.4	86.4
N1–Cr–N3	173.8	174.0	168.4
N1–Cr–N4	92.6	92.1	90.5
N1–Cr–N5	93.4	93.4	96.7
N1–Cr–N6	86.7	86.7	89.5
N2–Cr–N3	86.4	86.8	82.4
N2–Cr–N4	93.3	92.9	98.3
N2–Cr–N5	179.3	179.1	174.0
N2–Cr–N6	94.1	94.1	104.5
N3–Cr–N4	87.0	86.7	88.0
N3–Cr–N5	92.8	92.4	94.2
N3–Cr–N6	94.6	95.2	96.4
N4–Cr–N5	86.7	86.7	76.6
N4–Cr–N6	172.6	172.8	157.2
N5–Cr–N6	86.0	86.3	80.8
Cr–N2–C$_{para}$	179.4	179.4	160.2
Cr–N5–C$_{para}$	179.5	179.2	161.5

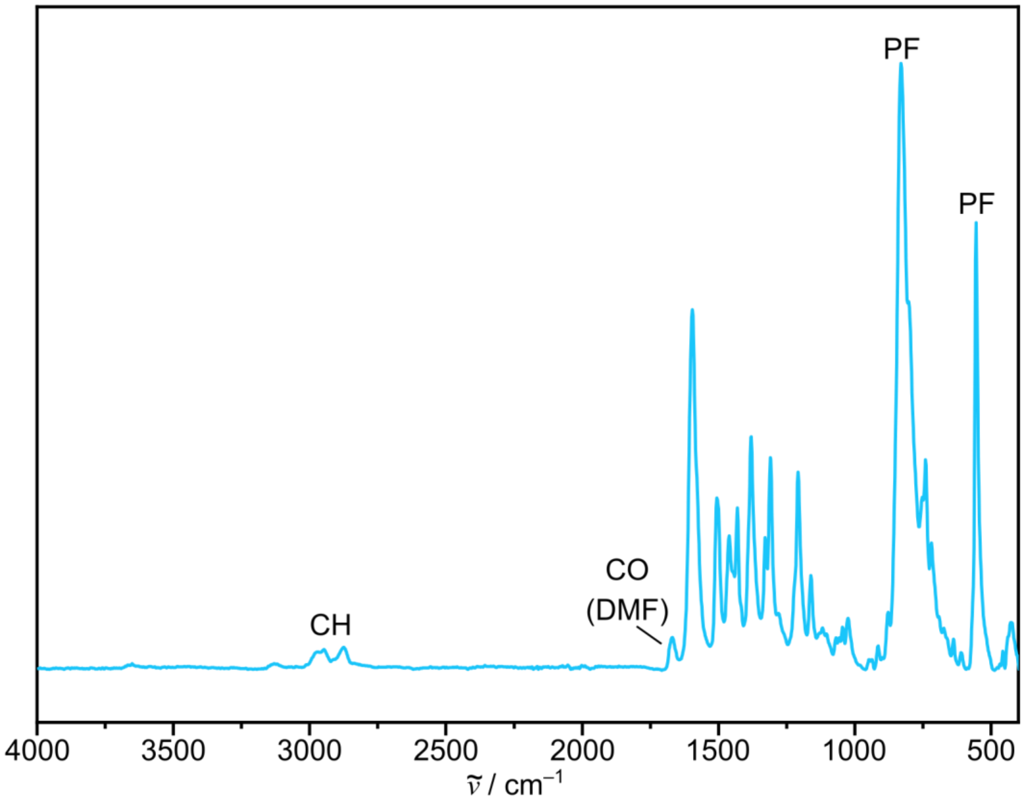

Figure S4. ATR-IR spectrum of **[Cr(dgpy)$_2$][PF$_6$]$_3$**. Some of the characteristic bands have been assigned.[1]

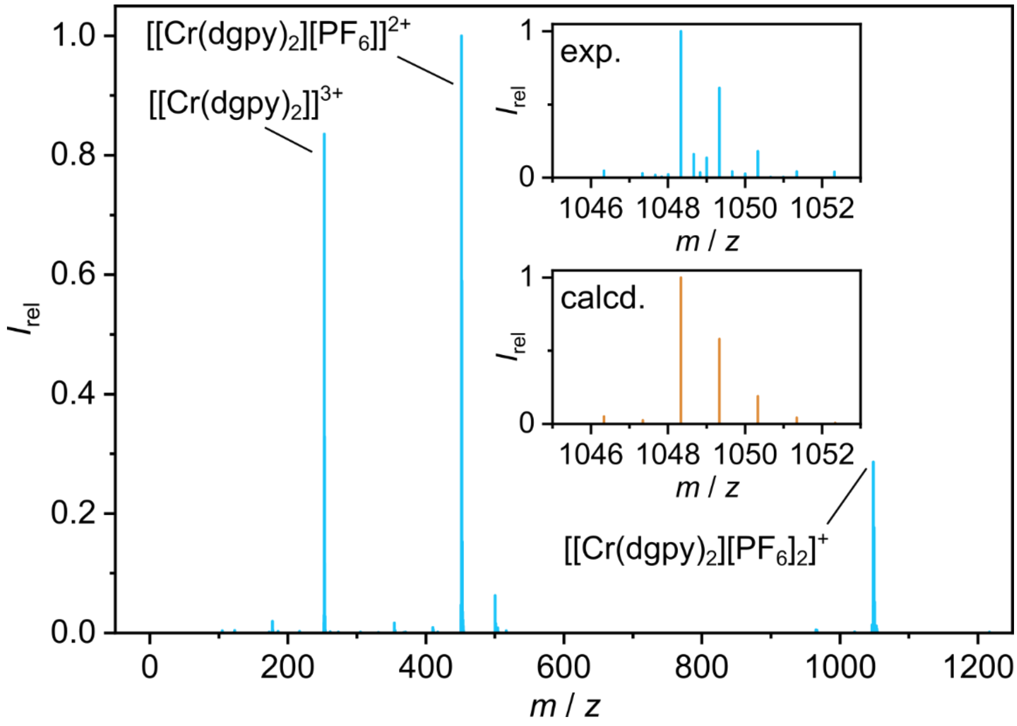

Figure S5. ESI$^+$ mass spectrum of **[Cr(dgpy)$_2$][PF$_6$]$_3$** in MeCN with comparison of experimental and calculated ($C_{38}H_{54}N_{14}F_{12}P_2Cr$) isotope patterns of the **[[Cr(dgpy)$_2$][PF$_6$]$_2$]$^+$** peak. The most intense peaks have been assigned.

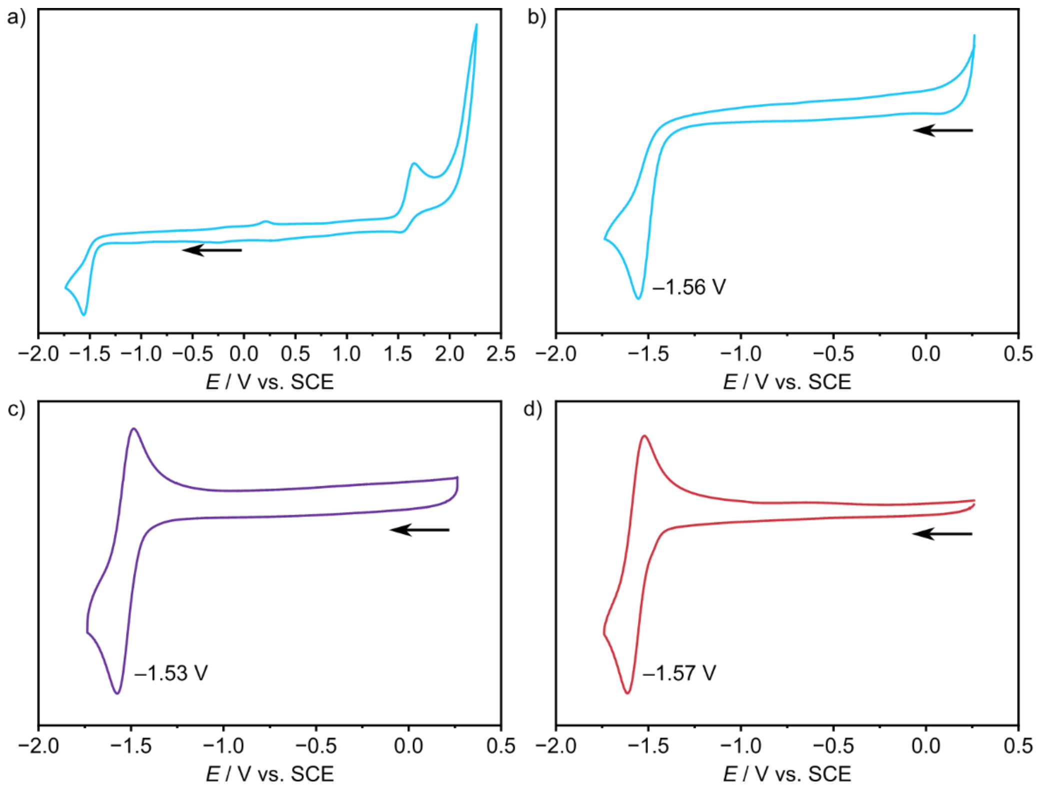

Figure S6. a/b) Cyclic voltammograms of 1 mM **[Cr(dgpy)$_2$][PF$_6$]$_3$** in a 100 mM solution of [*n*-Bu$_4$N][PF$_6$] in MeCN. The peak maximum has been labeled. c) Cyclic voltammogram of 1 mM **[Cr(dgpy)$_2$][OTf]$_3$** in a 100 mM solution of [*n*-Bu$_4$N][ClO$_4$] in MeCN. $E_{1/2}$ has been labeled. d) Cyclic voltammogram of 1 mM **[Cr(dgpy)$_2$][OTf]$_3$** in a 100 mM solution of [*n*-Bu$_4$N][ClO$_4$] in DMF. $E_{1/2}$ has been labeled.

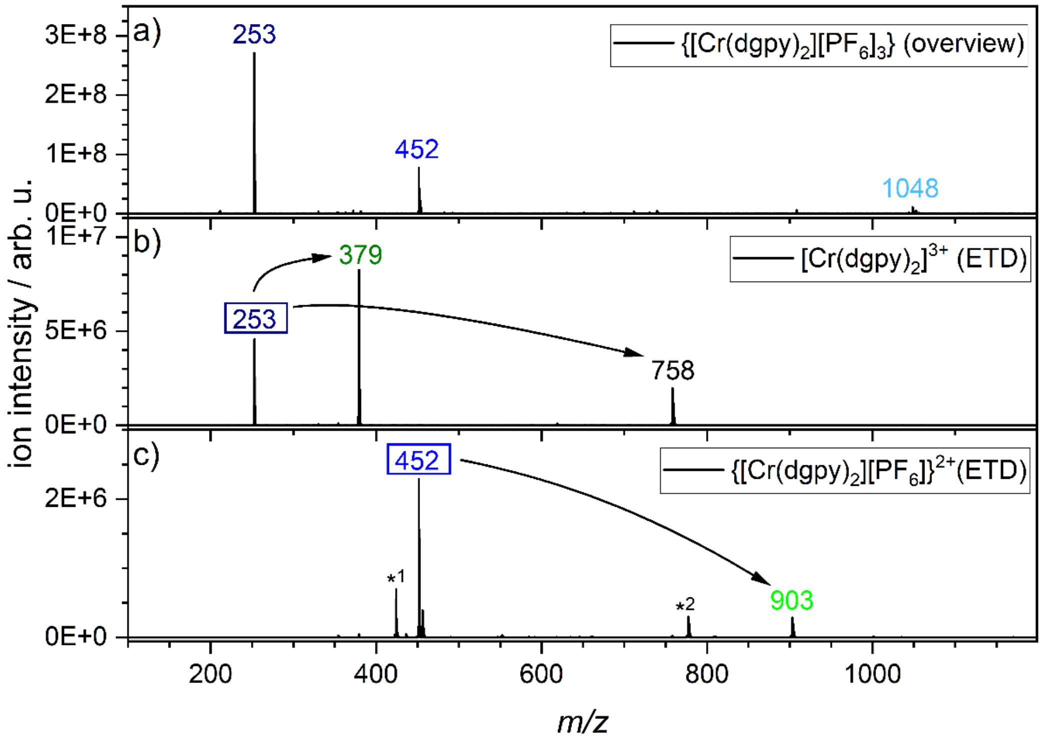

Figure S7. a) Overview mass spectrum of a solution of **[Cr(dgpy)$_2$][PF$_6$]$_3$** in MeCN. Resulting spectra of the ETD processes of b) **[Cr(dgpy)$_2$]$^{3+}$** and c) **{[Cr(dgpy)$_2$][PF$_6$]}$^{2+}$**. The arrows show one-electron reduction steps. The asterisks denote **{[Cr(dgpy)]F}$^+$** (*1) and **{[Cr(dgpy)$_2$]F}$^+$** (*2).

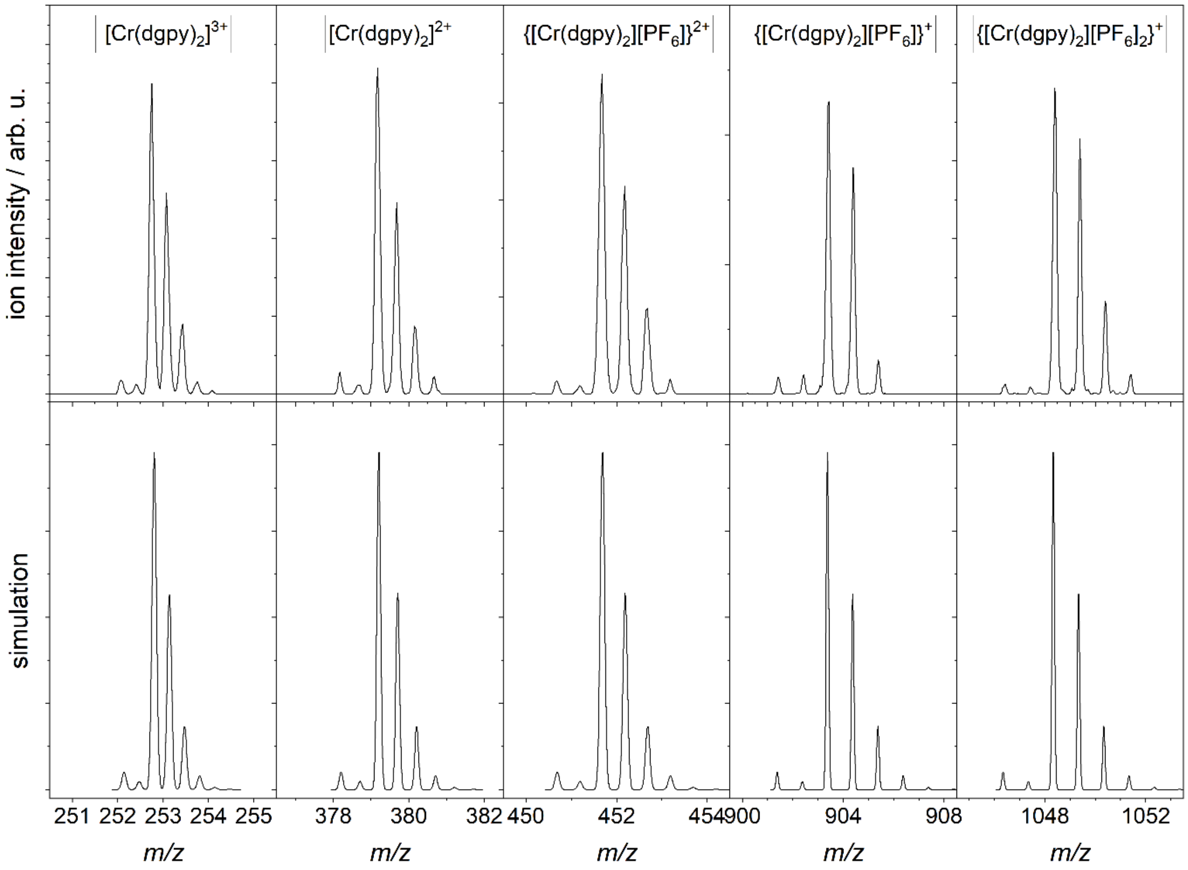

Figure S8. Mass spectra of the isolated species obtained from a solution of **[Cr(dgpy)$_2$][PF$_6$]$_3$** in MeCN in combination with ETD (top) and the respective simulated isotope patterns (bottom).

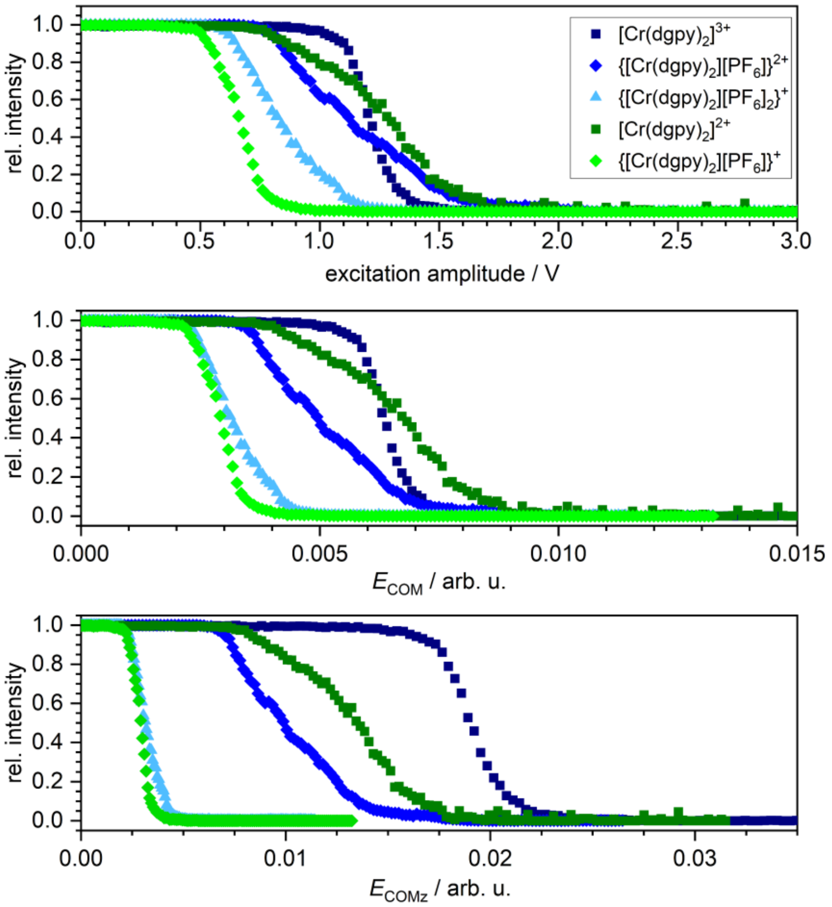

Figure S9. CID break down curves for the five investigated chromium species with different calculated energy axes. (top) the RF amplitude given by the software, (middle) mass corrected E_{COM} values to account for the different masses of the complex and (bottom) mass and charge corrected values E_{COMz} to account for the different charge states. Only the decrease of the parent ion is plotted against the mass and charge corrected E_{COMz} values (see method section).

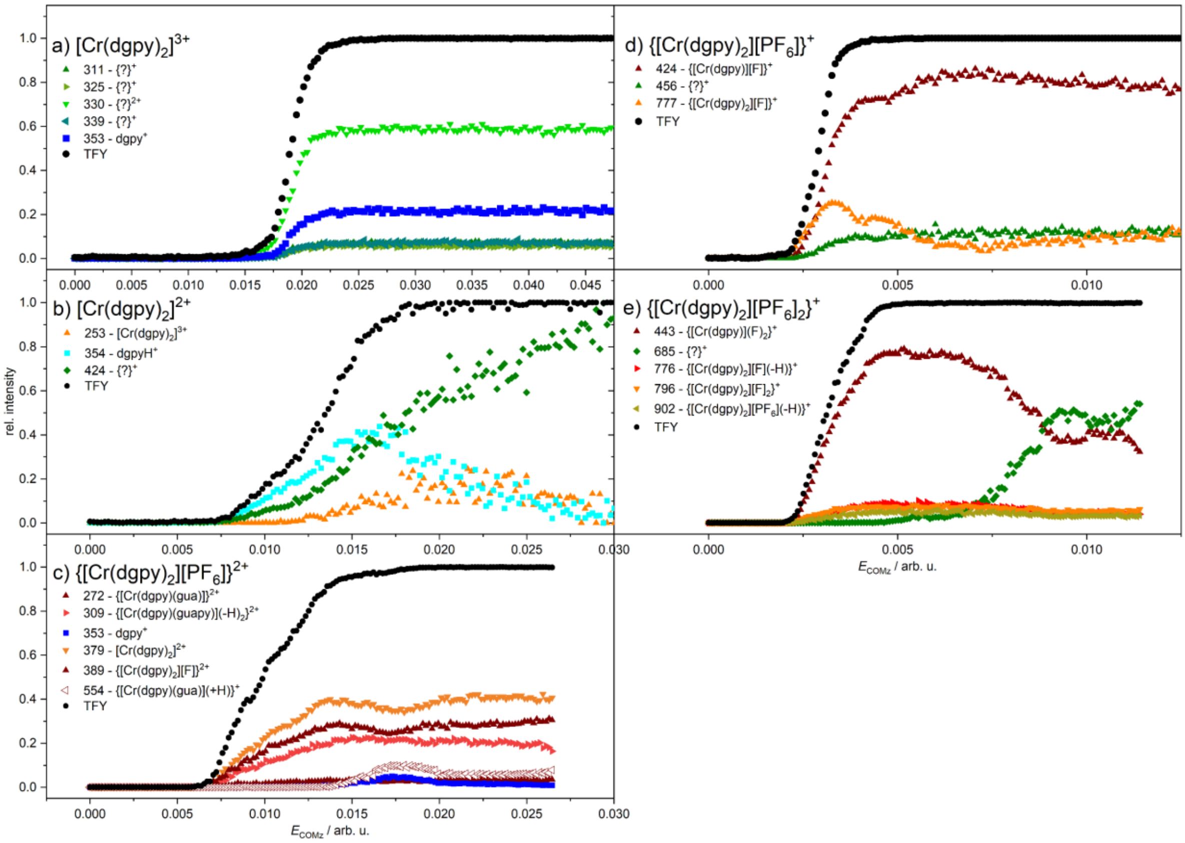

Figure S10. Comparison of the CID appearance curves against the respective E_{COMz} values of the chromium species a) **[Cr(dgpy)$_2$]$^{3+}$**, b) **[Cr(dgpy)$_2$]$^{2+}$**, c) **{[Cr(dgpy)$_2$][PF$_6$]}$^{2+}$**, d) **{[Cr(dgpy)$_2$][PF$_6$]}$^{+}$** and e) **{[Cr(dgpy)$_2$][PF$_6$]$_2$}$^{+}$**. Fragmentation channels are only assigned when reasonable molecular structures could be found.

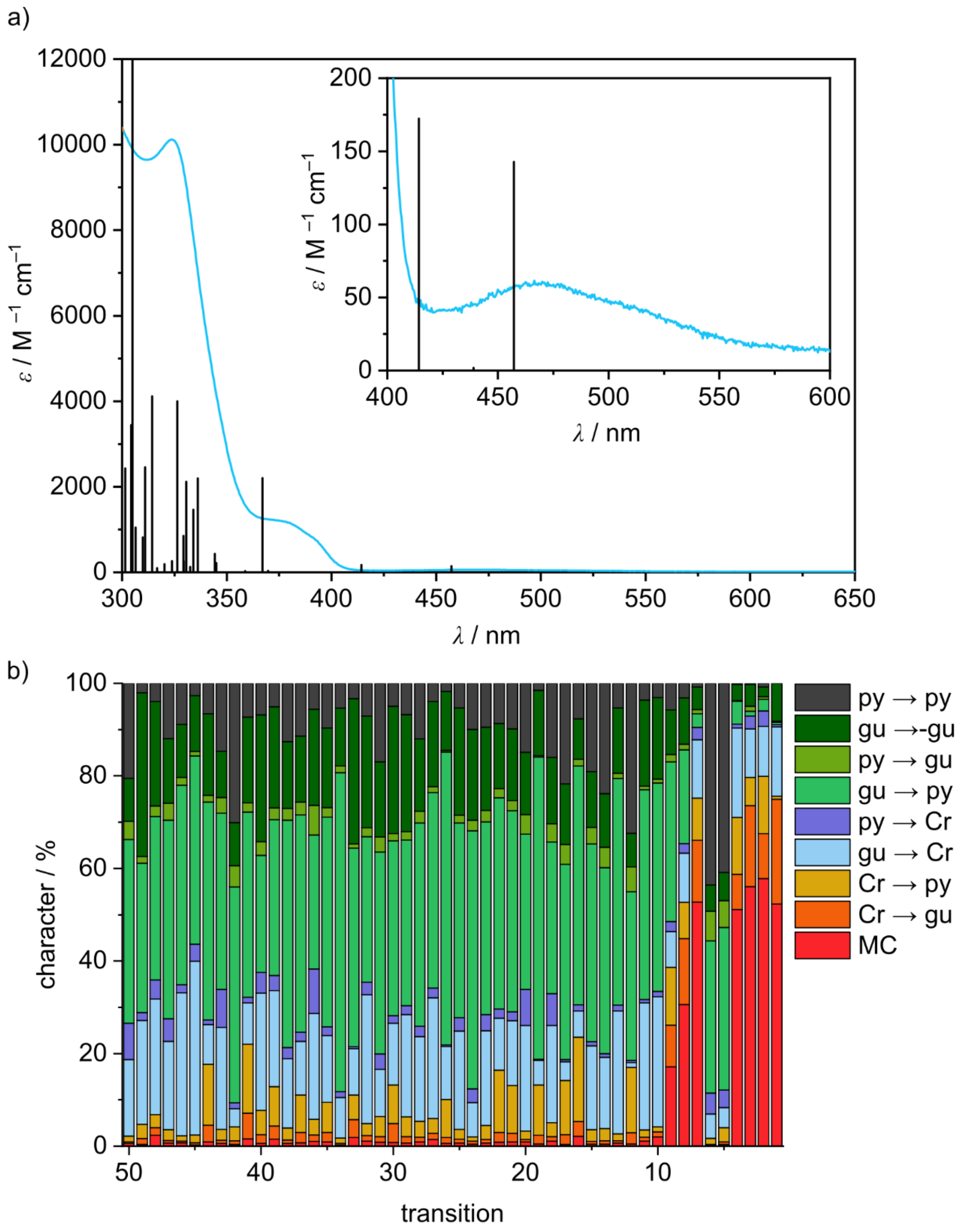

Figure S11. a) UV/vis absorption spectrum of **[Cr(dgpy)$_2$][PF$_6$]$_3$** in MeCN (blue). TD-DFT calculated transitions shown as vertical lines (black). b) Charge transfer number analysis of the 50 lowest energy spin-allowed transitions using the TheoDore software package.[1,2]

Table S2. TDDFT-calculated electronic transitions of **$[Cr(dgpy)_2]^{3+}$**. Orange: electron density gain, purple: electron density loss; hydrogen atoms omitted for clarity (CPCM(acetonitrile) ZORA SARC/J RIJCOSX B3LYP D3BJ ZORA-Def2-TZVPP).

#	λ / nm	f	TDDFT difference density (isosurface value 0.003 a.u.)
1	457.2	7.14×10^{-4}	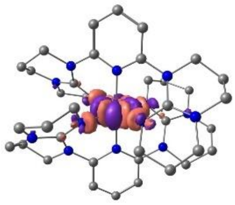
2	439.0	9.73×10^{-6}	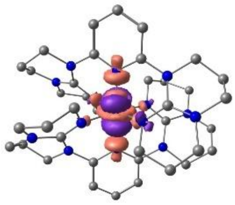
3	414.3	8.62×10^{-4}	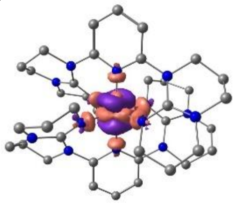
4	390.8	7.66×10^{-6}	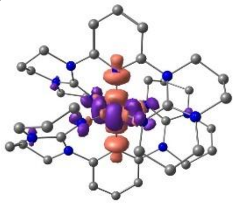
5	369.7	1.53×10^{-4}	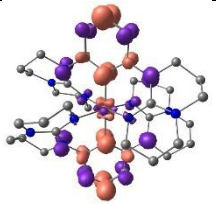
6	367.0	0.01099	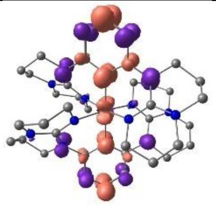
7	358.7	1.22×10^{-4}	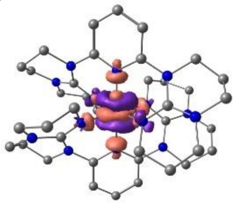
8	345.0	0.00109	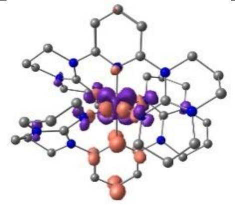

9	344.3	0.00216	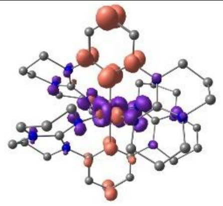
10	336.1	0.01098	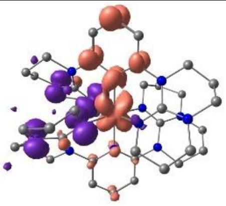
11	334.0	0.00732	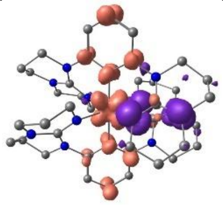
12	332.5	6.21 x 10^{-4}	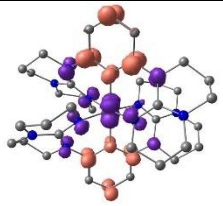
13	330.6	0.01059	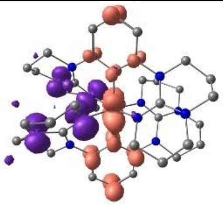
14	329.5	0.00132	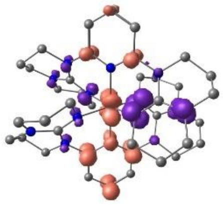
15	329.3	0.00425	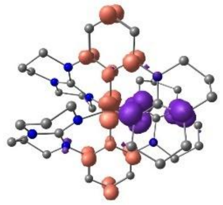
16	326.4	0.02	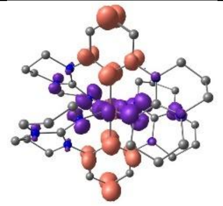
17	323.8	0.00131	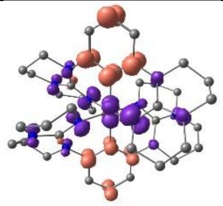

18	320.2	9.58×10^{-4}	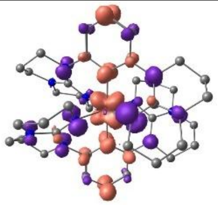
19	316.7	4.80×10^{-4}	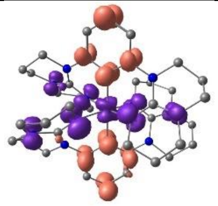
20	314.3	0.02058	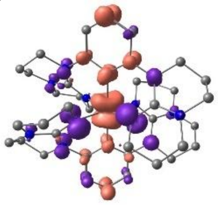
21	311.0	0.01229	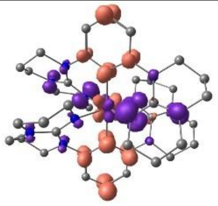
22	309.9	0.00408	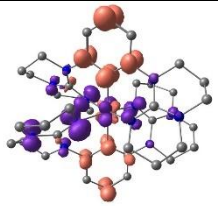
23	306.4	0.00522	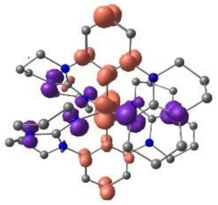
24	305.0	0.07363	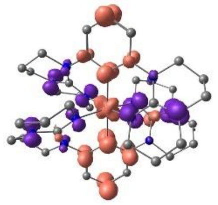
25	304.3	0.01722	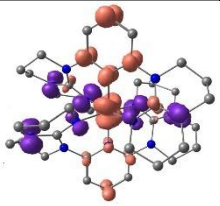
26	301.5	0.01215	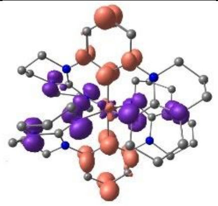

27	298.0	0.01081	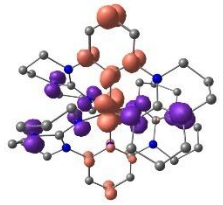
28	297.6	0.02873	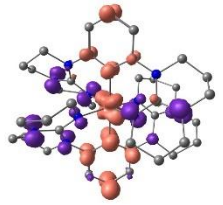
29	296.5	0.01494	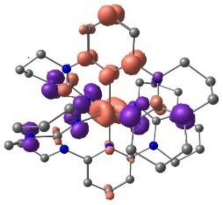
30	296.0	0.01239	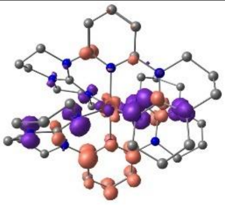
31	295.1	0.08182	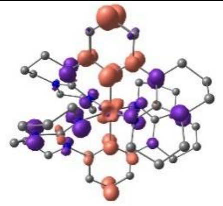
32	293.5	0.0143	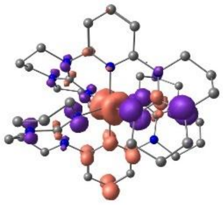
33	289.1	0.01106	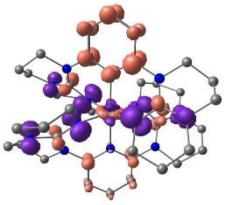
34	287.5	0.00225	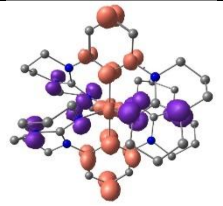
35	286.3	0.03436	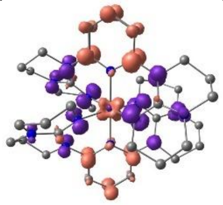

36	284.7	3.47 x 10^{-4}	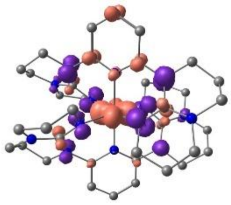
37	283.5	0.01567	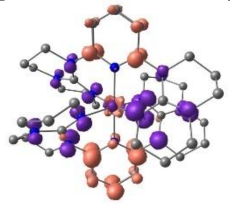
38	282.6	9.70 x 10^{-4}	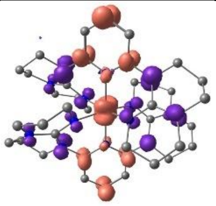
39	278.4	0.01384	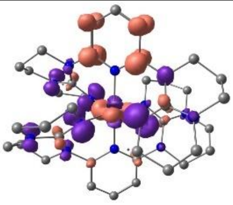
40	277.2	0.00314	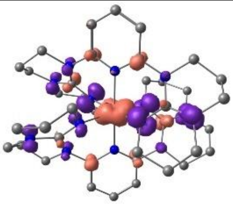
41	276.0	0.00157	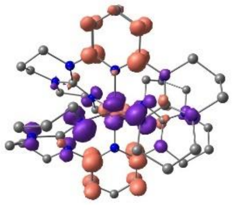
42	275.4	0.00129	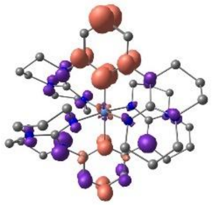
43	274.3	4.68 x 10^{-4}	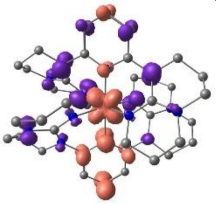
44	273.1	0.00244	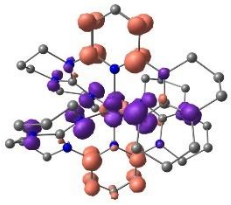

45	272.2	0.00158	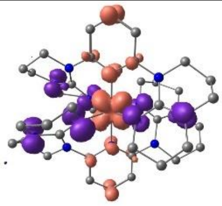
46	270.8	0.00349	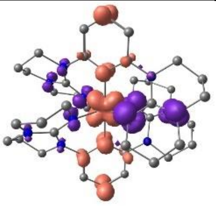
47	270.4	0.03952	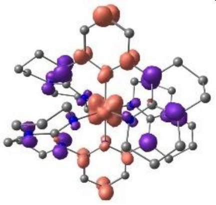
48	270.1	0.12339	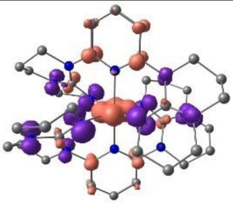
49	268.5	0.00305	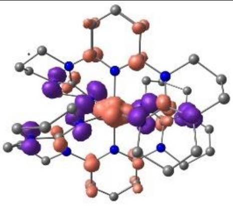
50	267.7	0.01959	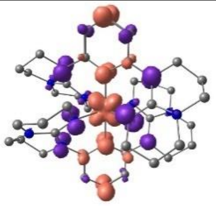

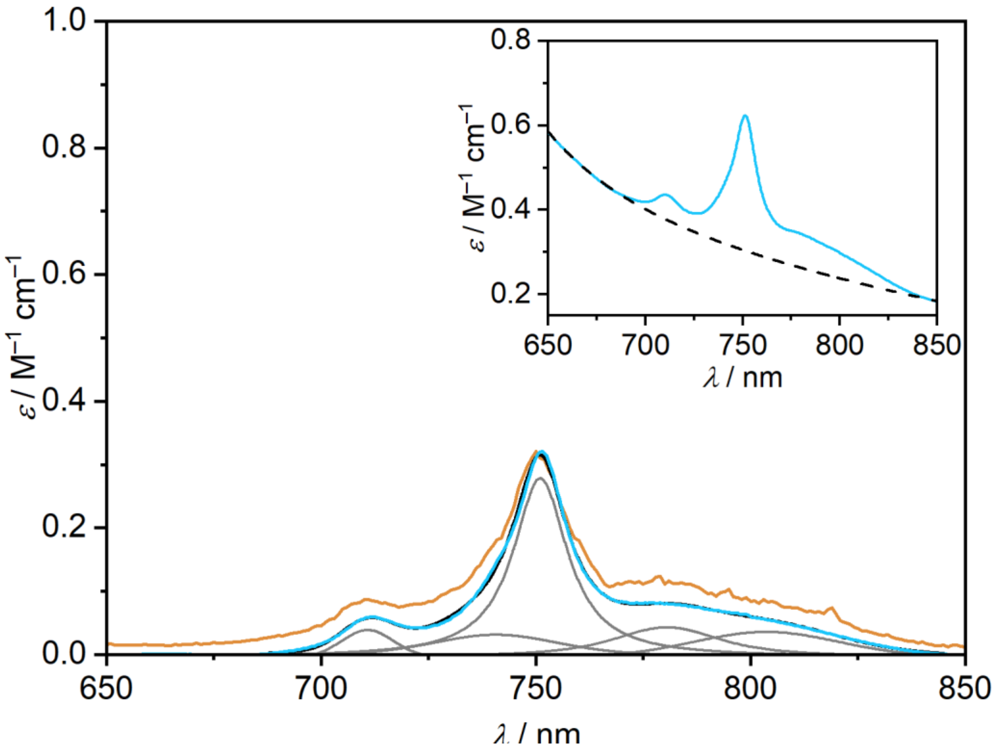

Figure S12. Baseline corrected vis/NIR absorption (blue) spectrum with sum fit (black) consisting of five Voigt functions (grey) and excitation spectrum (λ_{em} = 875 nm, orange) of **[Cr(dgpy)$_2$][PF$_6$]$_3$** in MeCN. Inset: vis/NIR absorption spectrum of **[Cr(dgpy)$_2$][PF$_6$]$_3$** (blue) with biexponential fit (black, dashed) describing the tail of the spin-allowed transitions as baseline correction.

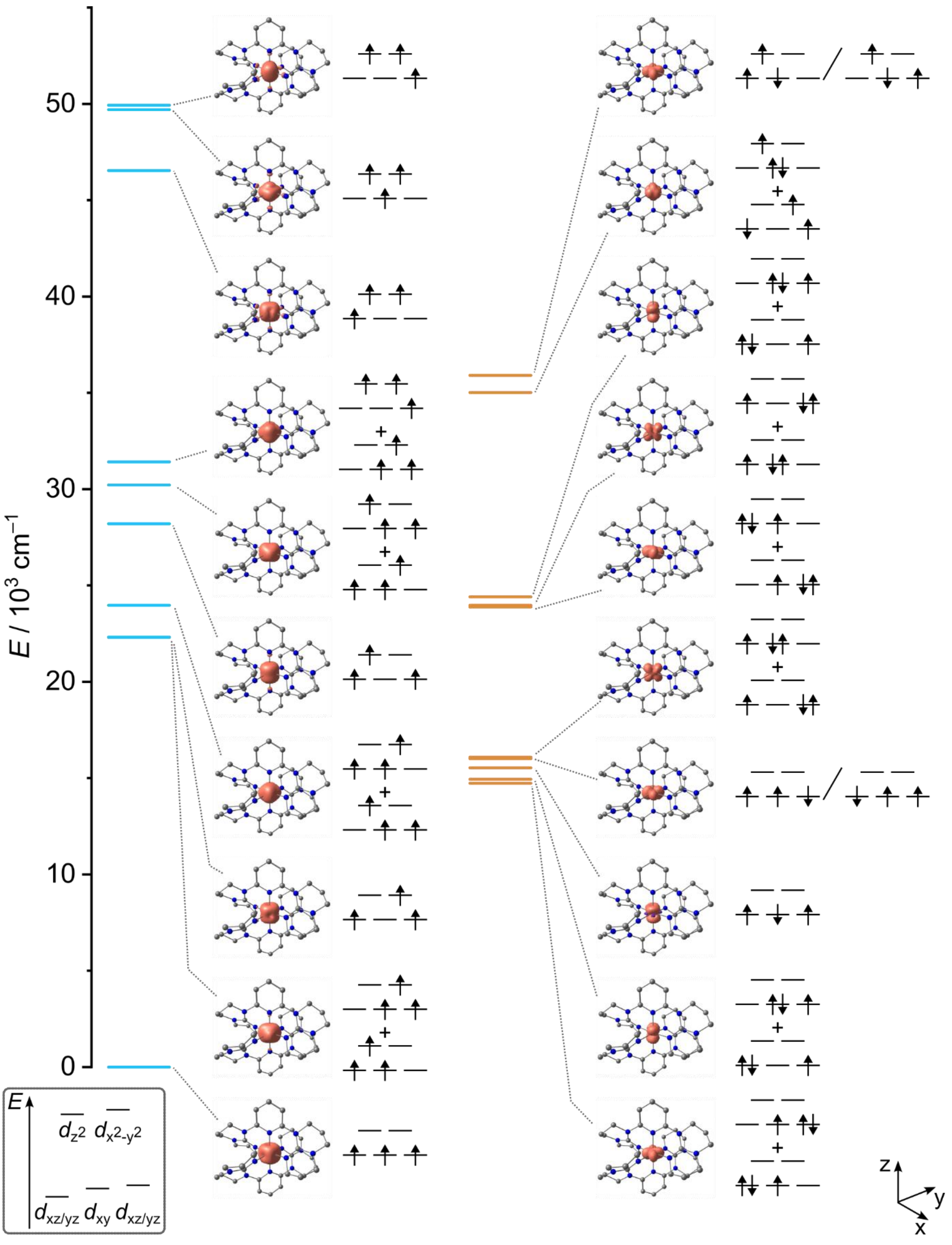

Figure S13. Energy levels of quartet (blue) and doublet (orange) states of **$[Cr(dgpy)_2]^{3+}$**, determined by CASSCF(7,12)-SC-NEVPT2 calculations (hydrogen atoms omitted for clarity). A coordinate system referring to the displayed structures and *d* orbital labels with their relative energies from the NEVPT2 calculations are included. Due to significant mixing, the d_{xz} and d_{yz} orbitals are labelled as $d_{xz/yz}$. Note that the orbital energies in the main figure do not reflect the symmetry lowering in order to avoid confusion, so that these levels merely serve to indicate the electron population.

Table S3. Energies and depictions of the orbitals used in the active space of the CASSCF(7,12)-SC-NEVPT2 calculation of **[Cr(dgpy)$_2$]$^{3+}$** (contour value of 0.03 a.u.; hydrogen atoms omitted for clarity).

#	*E* / H	orbital	#	*E* / H	orbital
197	–0.5621	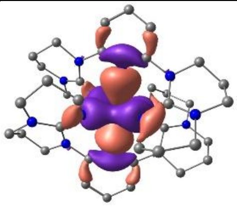	203	0.1761	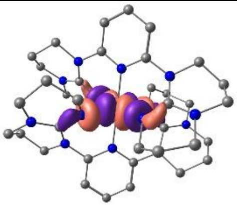
198	–0.5440	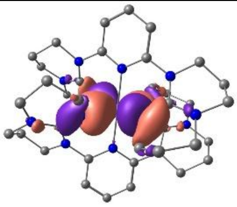	204	1.0338	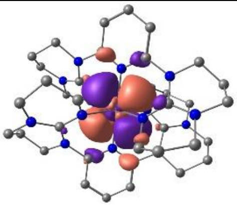
199	–0.1036	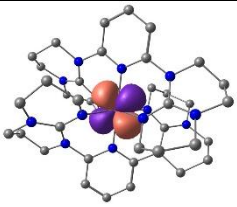	205	1.0509	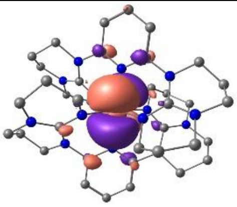
200	–0.0929	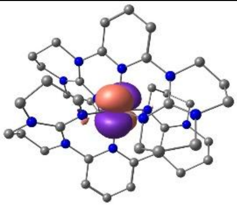	206	1.1155	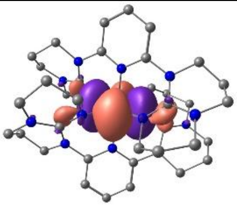
201	–0.0877	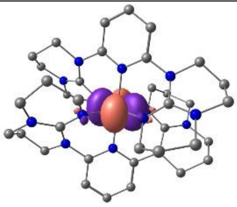	207	1.6130	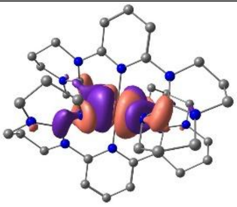
202	0.1753	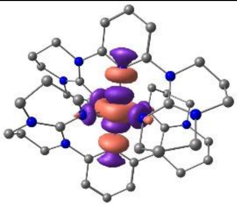	208	1.6952	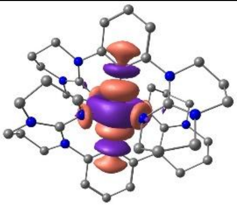

Table S4. CASSCF(7,12)-NEVPT results of $[Cr(dgpy)_2]^{3+}$; energies in cm^{-1} relative to the $^4A_2$ ground state (bold values indicate the lowest state of each multiplicity). Blue = quartet character, orange = doublet character. Doublet energies are scaled with 0.86 to better fit to the experiment in the third column.

Term symbol	*E* / cm^{-1}	*E* / cm^{-1} (scaled with 0.86)
**$^2T_1(1)$**	**14733.8**	**12671.1**
$^2T_1(2)$	14934.0	12843.2
$^2E(1)$	15529.8	13355.6
$^2E(2)$	16006.2	13765.3
$^2T_1(3)$	16080.0	13828.8
**$^4T_2(1)$**	**22302.8**	
$^4T_2(2)$	22318.3	
$^2T_2(1)$	23882.6	20539.0
$^2T_2(2)$	23962.9	20608.1
$^4T_2(3)$	23972.1	
$^2T_2(3)$	24401.3	20985.1
$^4T_1(1)$	28197.9	
$^4T_1(2)$	30219.1	
$^4T_1(3)$	31411.1	
$^2A_1$	35007.9	30106.8
$^2A_2$	35904.2	30877.6
$^4T_1(1)$	46532.2	
$^4T_1(2)$	49698.2	
$^4T_1(3)$	49923.1	

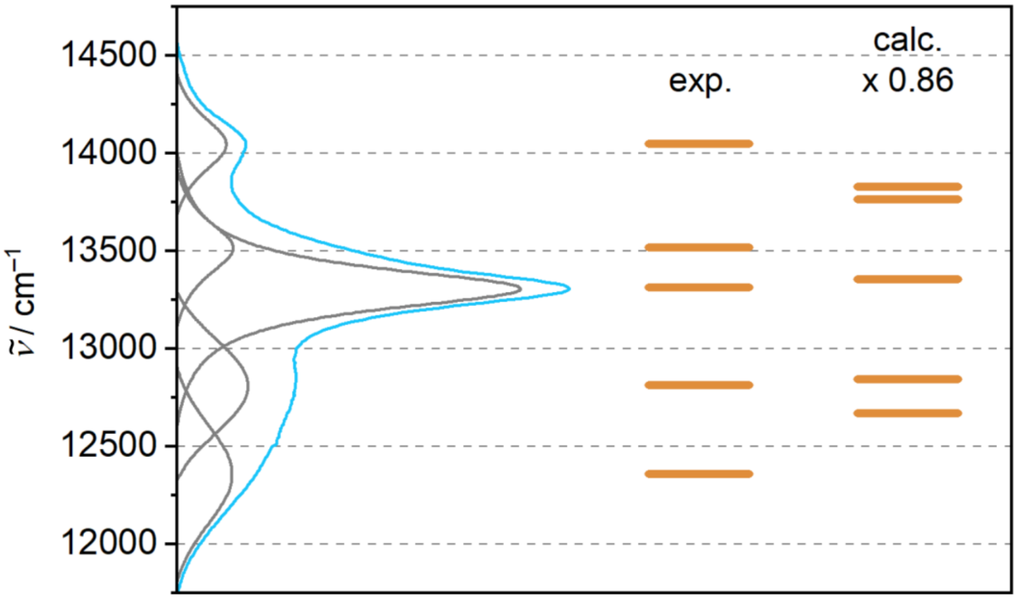

Figure S14. Comparison of experimental (from deconvoluted spin-flip absorption bands; exp.) and CASSCF(7,12)-SC/NEVPT2 calculated doublet energies (calc.). The calculated values were scaled with a factor of 0.86.

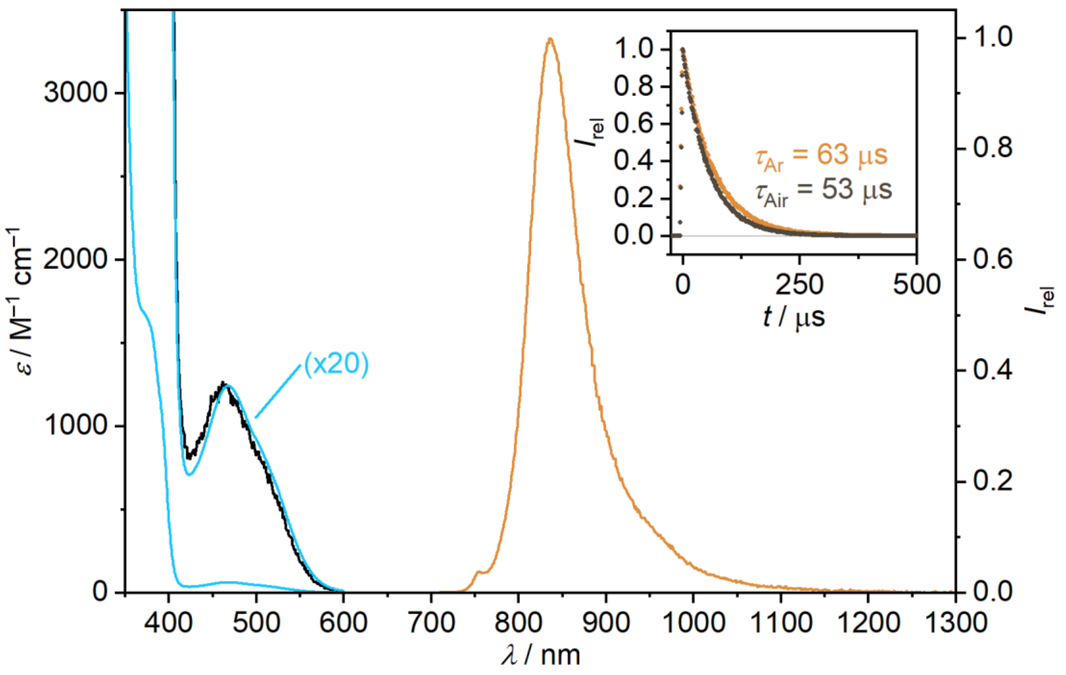

Figure S15. Absorption (blue), normalized excitation (λ_{em} = 836 nm, black) and emission (λ_{exc} = 468 nm, orange) spectra of **[Cr(dgpy)$_2$][PF$_6$]$_3$** in MeCN. Inset: emission decay traces recorded at 836 nm under argon (orange) and air (brown) after λ_{exc} = 451 nm.

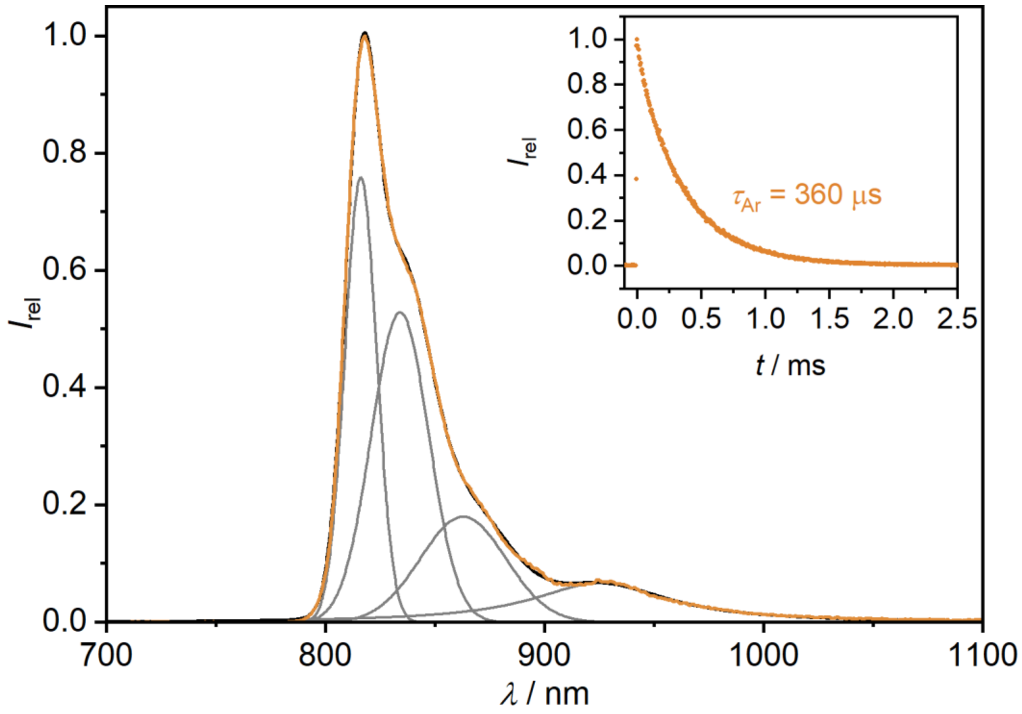

Figure S16. Normalized emission (λ_{exc} = 450 nm) spectrum of **[Cr(dgpy)$_2$][PF$_6$]$_3$** in *n*-PrCN at 77 K with sum fit (black) consisting of four Voigt functions (grey). Inset: emission decay trace recorded at 836 nm under argon.

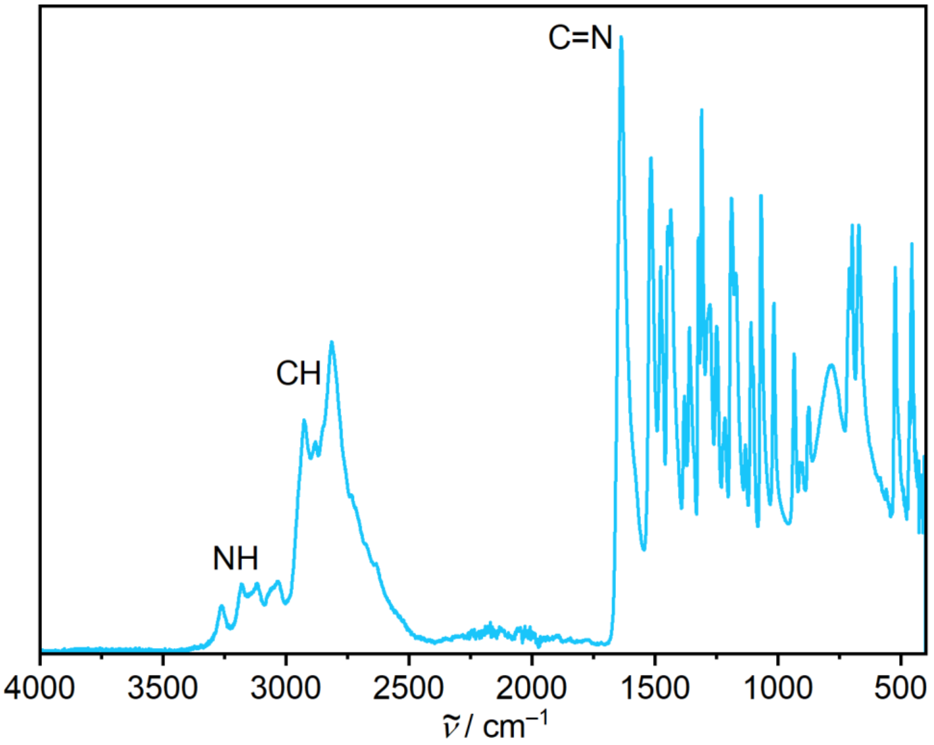

Figure S17. ATR-IR spectrum of 1,5,7-triazabicyclo[4.4.0]dec-5-ene. Some of the characteristic bands have been assigned.[3,4]

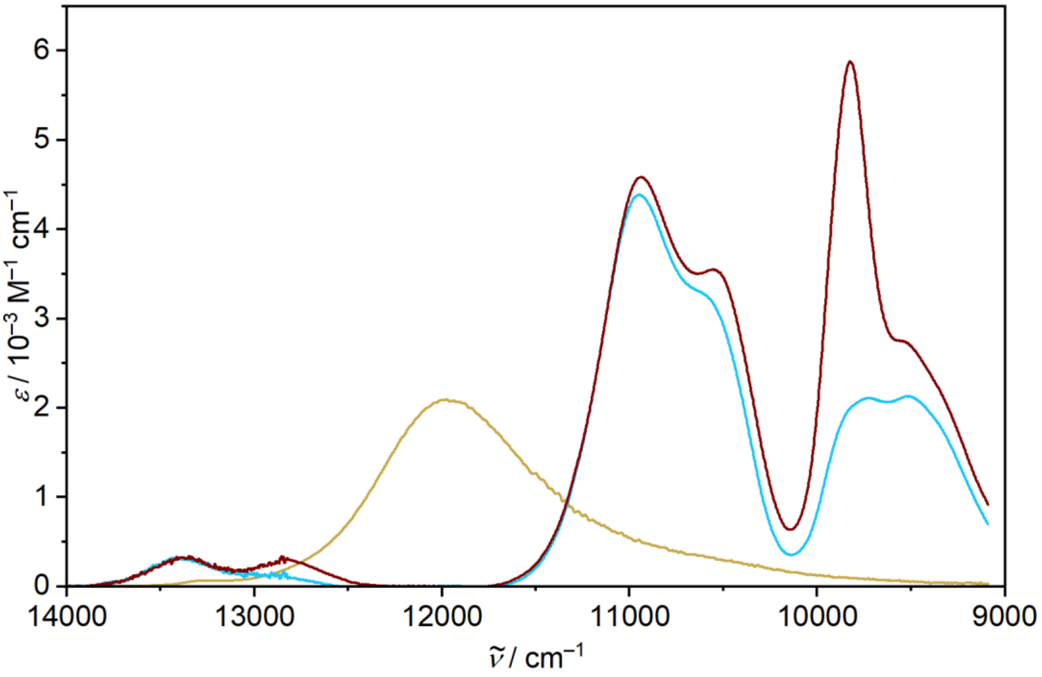

Figure S18. NIR absorption spectrum of non-deuterated (brown) and *N*-deuterated (blue) 1,5,7-triazabicyclo[4.4.0]dec-5-ene in CD_2Cl_2 overlaid with the emission spectrum (normalized to a unit area) of **[Cr(dgpy)$_2$]$^{3+}$** in MeCN at 293 K (orange).

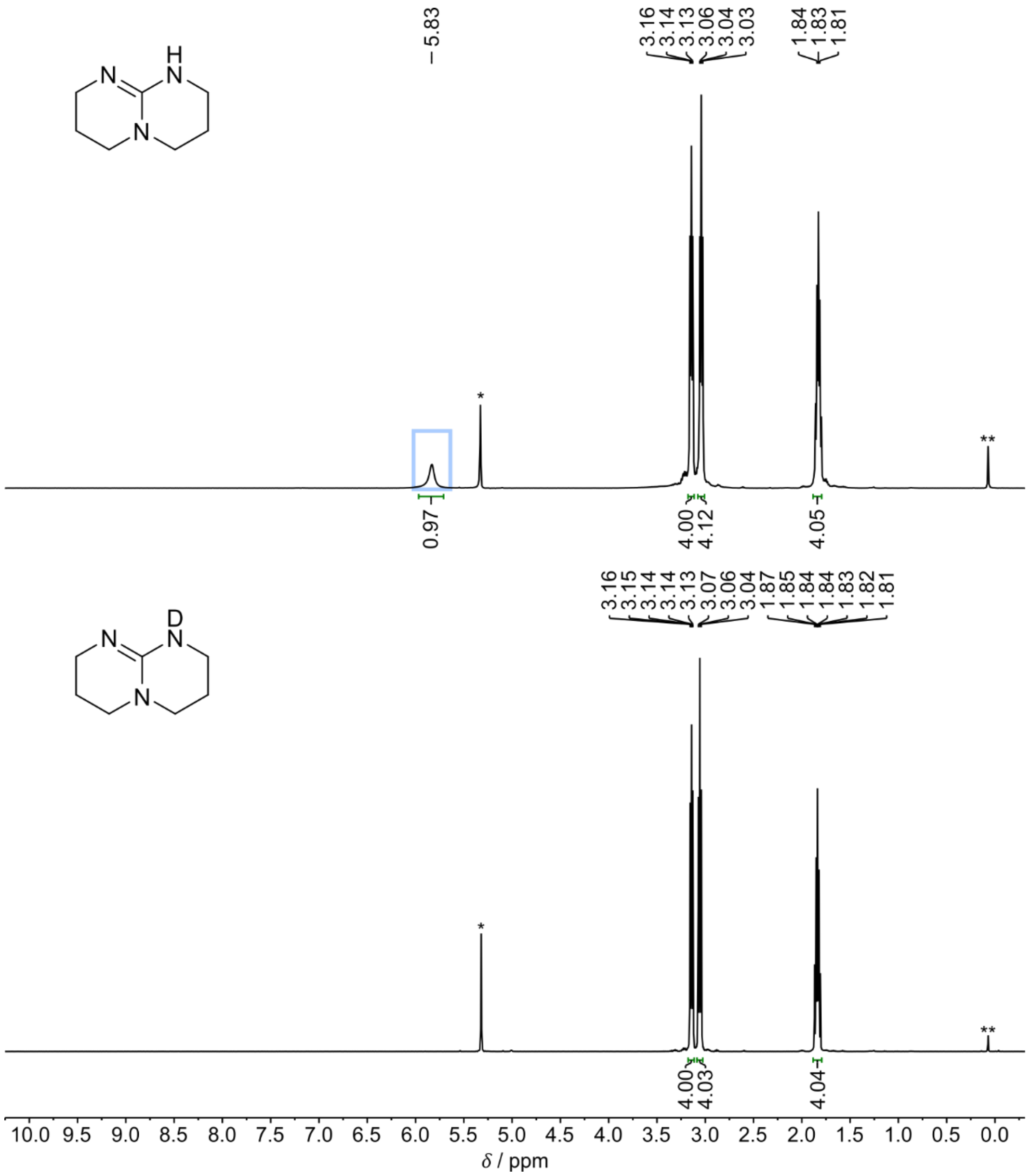

Figure S19. ^{1}H NMR spectra of 1,5,7-triazabicyclo[4.4.0]dec-5-ene in CD_2Cl_2 before and after *N*-deuteration. The N-H proton resonance of the non-deuterated molecule is highlighted in blue. The asterisk * denotes dichloromethane solvent resonance. The double asterisk ** denotes silicone grease resonance.

References

(1) Mai, S.; Plasser, F.; Dorn, J.; Fumanal, M.; Daniel, C.; González, L. Quantitative wave function analysis for excited states of transition metal complexes. *Coord. Chem. Rev.* **2018**, *361*, 74–97. DOI: 10.1016/j.ccr.2018.01.019.

(2) Plasser, F. TheoDORE: A toolbox for a detailed and automated analysis of electronic excited state computations. *J. Chem. Phys.* **2020**, *152*, 84108. DOI: 10.1063/1.5143076.

(3) Larkin, P. J. *Infrared and Raman Spectroscopy*; Elsevier Inc., 2018. 2nd. ed., DOI: 10.1016/C2015-0-00806-1.

(4) Brzezinski, B.; Schroeder, G.; Rybachenko, V. I.; Kozhevina, L. I.; Kovalenko, V. V. Study of 1,5,7-triazabicyclo[4,4,0]dec-5-ene protonation by vibrational spectroscopic methods. *J. Mol. Struct.* **2000**, *516*, 123–130. DOI: 10.1016/S0022-2860(99)00129-5.
